# Mesoporous nanomaterial-based smart nanoplatforms for precise therapies of ulcerative colitis: Strategies and challenges

**DOI:** 10.7150/thno.109268

**Published:** 2025-07-11

**Authors:** Zihong Xie, Jun Wu, Jin Xie, Hongxin Chen, Xinmin Wang, Junfeng Huang, Baode Shen, Hao Song, Lina Fu, Pengfei Yue

**Affiliations:** 1 State Key Laboratory of Modern Preparation of Classical and Famous Prescriptions of Chinese Medicine, Jiangxi University of Chinese Medicine, Nanchang 330004, China.; 2 Department of Pharmaceutics, 908th Hospital of Joint Logistics Support Force of PLA, Nanchang 330000, China.; 3 Pharmacy Department, University of Kansas, Lawrence, KS 66045, USA.; 4 Australian Institute for Bioengineering and Nanotechnology, the University of Queensland, Brisbane QLD 4072, Australia.; 5 Pharmacy Department, Shanghai Eighth People's Hospital, Shanghai, China.; Zihong Xie and Jun Wu contributed equally to this work.

**Keywords:** mesoporous nanomaterials, ulcerative colitis, smart nanotherapies, drug delivery

## Abstract

Ulcerative colitis (UC) is a chronic inflammatory disease predominantly impacting the rectum and colon. Because UC has a complicated pathophysiology, there is no effective treatment so far. Over the past decade, nanomedicine has undergone a remarkable transformation and expansion in its application, driven by an increasingly profound comprehension of the multifaceted functions of diverse nanomaterials. Consequently, an increasing number of these advanced materials have been utilized in the treatment of UC. Notably, mesoporous nanomaterials (MNMs) have gained significant attention due to their unique physicochemical properties, including high specific surface area, tunable pore size, large pore volume, and customizable surface chemistry. These MNMs exhibit outstanding drug-loading capacity, controlled release behavior and excellent biocompatibility, which have been demonstrated successfully overcoming the limitations of conventional therapies. With increasing research interest and translational potential, this review highlights novel strategies for UC treatment by focusing on its physiopathological characteristics and summarizes recent advancements in applying MNMs for UC therapy. The design strategies of various MNM-based smart nanoplatforms as drug carriers for UC treatment are comprehensively reviewed, with critical challenges and future trends in the development and translation of MNMs for UC therapies also outlined. This review establishes a rational framework for designing smart drug delivery systems based on mesoporous nanomaterial and provides guidance and inspiration for UC and other inflammatory disease treatment.

## Introduction

Ulcerative colitis (UC) is defined by chronic, nonspecific inflammation of the colonic and rectal mucosa. Over the past few decades, there has been a notable increase in the global incidence of UC 1. Furthermore, the World Health Organization (WHO) has formally classified UC as a medically refractory disease. Conventional treatments, including aminosalicylates, glucocorticoids, immunosuppressants, and biologics, primarily alleviate symptoms but may cause side effects and have limited efficacy 2. This limitation can be attributed to the anatomical location of colonic lesions in the digestive tract's distal portion, which presents substantial obstacles for drugs to effectively reach the target sites. To address these issues, more and more efforts are focusing on developing advanced nanocarriers to load anti-ulcerative colitis drugs, thereby constructing drug delivery systems (DDSs) that enhance targeting, bioavailability, and therapeutic efficacy while minimizing side effects.

Smart nano-drug delivery systems (SNDDs) are generally advanced drug delivery nanoplatforms integrating sensing, processing and execution functions using functional nanomaterials together with drugs, which aims to deliver drugs to the lesion and then judge whether to release them based on stimulation signals, thereby achieving precise on/off control of drug release. Besides, SNDDSs have many unique advantages in the targeted treatment of UC, such as for the positively charged colonic microenvironment of UC patients, SNDDSs can be surface-modified to confer a negative charge on the drug, thus achieving a better targeting effect and reducing the side effects of systemic absorption; for some drugs that are degrade easily in the upper gastrointestinal tract (GIT), SNDDSs can encapsulate drugs within specific responsive systems (e.g., pH, enzyme, temperature), enabling the drugs to traverse the harsh upper gastrointestinal tract (GIT) environment and reach the colonic target site, thereby significantly enhancing drug bioavailability. Most importantly, SNDDSs can facilitate personalized treatment by adjusting the drug release rate and dosage according to individual patient conditions, which is not possible for conventional DDSs.

Over the past decades, a wide range of nanomaterials have been employed in SNDDSs. Among all the carriers, the porous nanomaterials have become a focus of increasing interest owing to their customizable physicochemical properties, which endow them with exceptional adaptability for a variety of applications. According to pore sizes, porous materials are divided into three types by the International Union of Pure and Applied Chemistry (IUPAC): microporous (pore size < 2 nm), mesoporous (2-50 nm), and macroporous (> 50 nm) 3. Compared with conventional nanocarriers (e.g., liposomes, polymeric nanoparticles, micelles), MNMs possess the characteristics of large specific surface area, tailorable pore size, diverse surface chemistry, customizable framework composition, and excellent biocompatibility. These paramount features make MNMs ideal nanoplatforms with multi-functionalities for treatment of UC, particularly in achieving high payload, drug targeting and smart release 4-6. For example, Manzano *et al.* systemically reviewed the synthesis strategies of mesoporous silica nanoparticles, their capacity for loading anti-inflammatory drugs, intelligent control of drug release, and their applications in UC 7. Although numerous studies have reported targeted drug delivery mediated by MNMs, the essential design and delivery strategies that apply MNMs into smart nanoplatforms for UC treatment have not been adequately explored.

**Figure 1:**
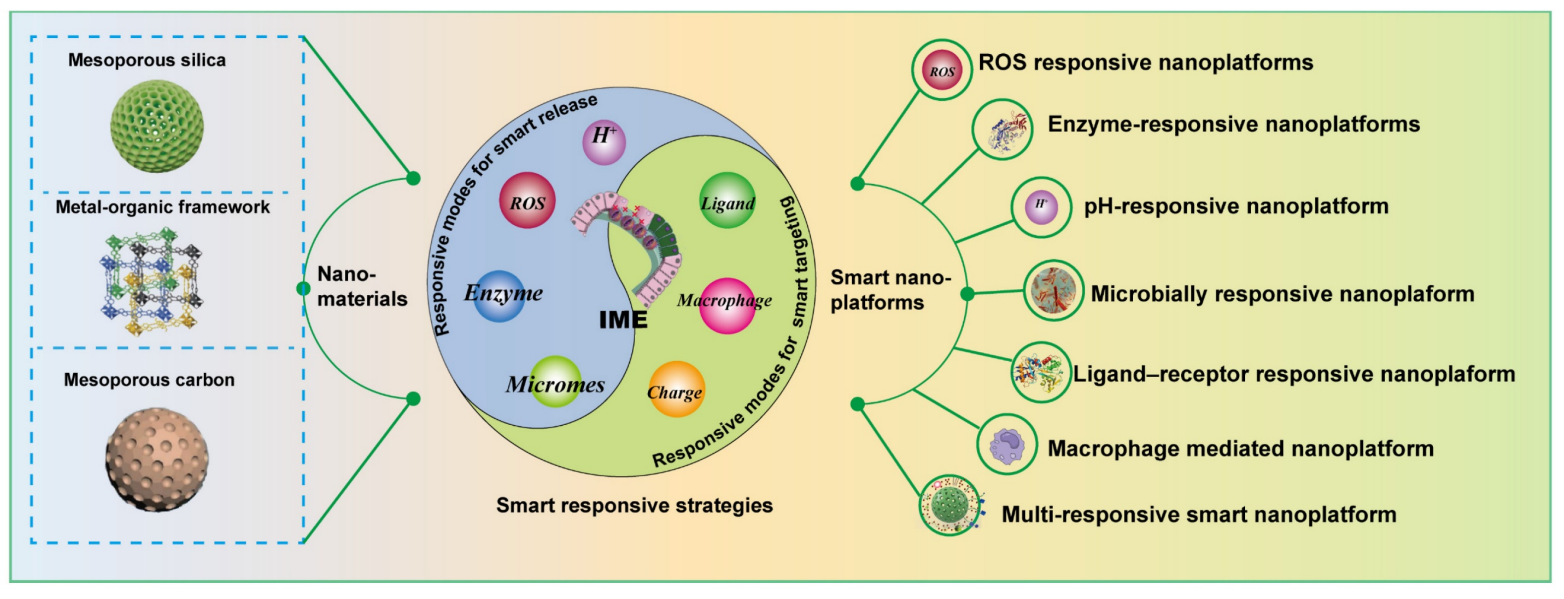
Schematic representation of mesoporous nanomaterials for smart drug delivery.

Therefore, this review addresses the topic from an underexplored perspective, shedding light on MNMs-based smart nanoplatforms for the management of UC. As displayed in (**Figure 1**), we first provide a comprehensive summary on the pathological environment of UC as well as the physiological barriers that must be overcome in designing UC therapies. We highlight recent advancements in MNMs that have been used as smart nanocarriers for UC treatment, including mesoporous silica nanoparticles (MSNs), metal-organic frameworks (MOFs), and mesoporous polydopamine (MPDA), etc. In addition, a comprehensive discussion was also presented on versatile design strategies of MNMs-based smart nanoplatforms for UC treatment. Beyond single responsiveness, the integrated nanotherapies combining various therapeutic approaches have been further discussed. Finally, we analyze the challenges associated with MNMs-based smart responsive nanoplatforms for UC treatment. The aim of this review is to provide insights that may inspire and guide further research and development of MNMs-based smart nanoplatforms for precise treatment of UC.

## Pathophysiology and Barrier of Ulcerative Colitis

### Pathophysiology of ulcerative colitis

The microenvironment surrounding colonic region in UC patients possesses distinct characteristics. In terms of its pathophysiology, UC appeared to result from an abnormal immune response directed against commensal and harmless bacteria, which occurred when the host's mucosal immune system was dysregulated or out of balance with the intestinal microbiota. Furthermore, in UC site, the colonic microenvironment becomes positively charged, marked by a significantly decreased pH, elevated levels of reactive oxygen species (ROS), and heightened concentrations of inflammatory cytokines, including interleukins (IL-5 and IL-13) 8. There is significant expression of certain receptors within the colonic mucosa of individuals with UC. Tumor necrosis factor (TNF) and IL-1β are more readily produced by neutrophils in UC, leading to increased accumulation and deployment of chromatin-based extracellular traps at colonic mucosal damage sites and thereby prolonging inflammation 9. In addition to macrophages, dendritic cells present antigens and promote immunological tolerance to environmental stimuli. They also direct T-cell differentiation to elicit adaptive immune responses. Pro-inflammatory cytokine production and less tolerant subpopulations of dendritic cells with a decreased ability to produce regulatory T cells are observed in the inflamed colon 10. Furthermore, the expression of all four members of the JAK family is significantly upregulated in active UC, including tyrosine kinase 2 (TYK2), JAK1, JAK2, and JAK3. Notably, JAK3 is absent in healthy intestinal mucosa but exhibits marked upregulation in a specific subpopulation of inflammatory fibroblasts 11. As the condition of UC deteriorates, on colonic epithelial cells and macrophages, the expression of specific receptors or cell adhesion molecules rises, creating possible binding sites for the personalization of drug delivery systems 12, 13. Commensal bacteria from the phyla *Firmicutes phyla* and *Bacteroidetes* were depleted in UC, according to surgical specimens that examined the gut wall microbiome 14. Similarly, other investigations have found decreased abundances of the butyrate-producing species *Faecalibacterium prausnitzii* and *Roseburia hominis* from the *Firmicutes phylum* 15. Butyrate exhibits antibacterial and anti-inflammatory effects and serves as a critical energy source for colonocytes 16. Several researchers have reported success in managing UC by restoring the balance of microbiota via the transplantation of microbes sourced from healthy donors. Nevertheless, a comprehensive understanding of the precise therapeutic mechanisms underlying this treatment approach is crucial for aligning it with contemporary therapeutic strategies.

**Figure 2:**
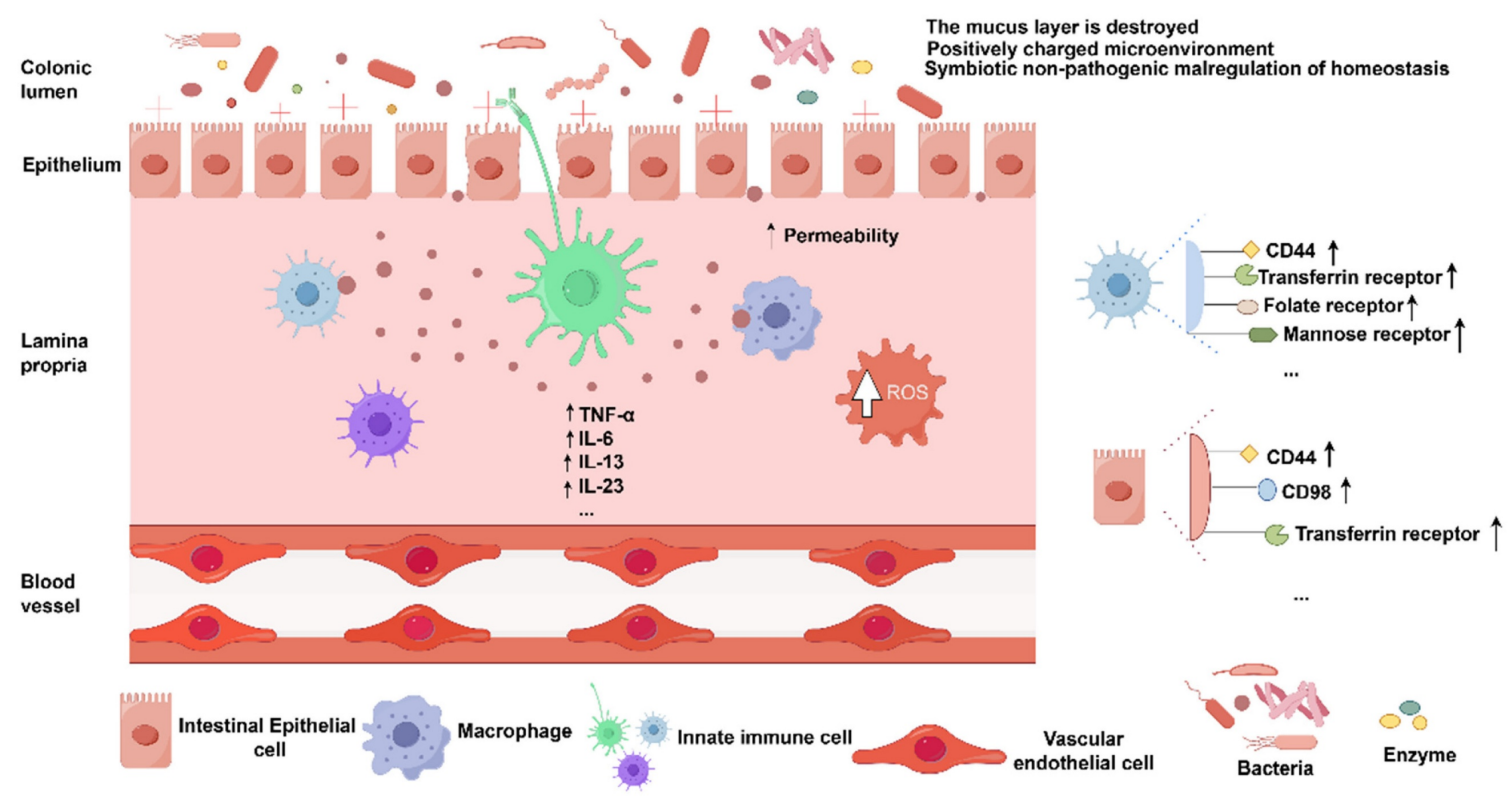
Characteristics of pathological microenvironment of UC. Imaged by Figdraw.

To effectively treat UC, the physiological characteristics of the colon must be taken into account, together with the distinctions between an inflammatory colon and a healthy peripheral milieu. These include changes in drug transit time, pH conditions, mean colonic fluid volume, enzyme content, microbiota species, and mucus layer thickness under UC 17. In addition, interindividual differences in food content also might influence the efficiency of medication transport to the colon and ought to be taken into account when designing colon-targeted systems. Ideally, to achieve optimal colon-targeted therapeutic effects, it is crucial to ensure that the drug remains stable and unabsorbed as it traverses the harsh chemical and enzymatic environment of the upper gastrointestinal tract (GIT), is not degraded by enzymatic activity, and is released exclusively at the specific sites of inflammation in the colon, thereby minimizing systemic toxicity and enhancing therapeutic efficacy. The pathophysiology of UC is illustrated in (**Figure 2**).

#### Defective gut epithelial barrier

By separating host immune cells from intestinal bacteria and promoting the production of antimicrobial peptides, the mucus layer, the outermost layer of the epithelial barrier, acts as the mucosal immune system's main line of defense. Colonic mucus, primarily composed of mucin-2 (MUC-2), forms a protective barrier by being released by goblet cells, however there are less of these cells in UC 18, 19. The barrier function's integrity is further compromised by apoptosis and the upregulation of tight junction proteins, which are critical paracellular permeability-regulating components of apical tight junctions 20. Defective regulation of tight junction proteins leads to increased permeability, though it is unclear, whether these findings are causal or associative 21.

In order to strengthen host defenses and limit bacterial invasion, the intestinal epithelium also produces antimicrobial peptides, such as defensins. Certain human beta-defensins have increased expression in colonic samples from UC patients. However, it is still unknown if inflammatory cytokines, microbes, or both are responsible for this rise in defensin synthesis 22.

#### Commensal microbiota

The regulation of gut health is significantly influenced by the gut flora. The gut microbiota and host in healthy people continue to coexist in a symbiotic relationship. They carry out a number of vital and special tasks, such as metabolic processes, immune-modulatory effects, preserving the mucus barrier by avoiding pathogen damage, and improving epithelial integrity 23. Many underlying factors, including genetic factors such as issues with autophagy, antimicrobial peptides, bacterial management, and cytokines, as well as environmental factors like smoking, anxiety, infections, overuse of antibiotics, vitamin D deficiency, and diets that alter the microenvironment of gastrointestinal epithelial cells, play crucial roles in triggering intestinal inflammation and impairing the body's immune tolerance mechanisms as imbalances in the intestinal microbiota and commensal bacteria become pathogenic 24. Normally, the intestinal immune system plays a role in constraining and regulating the intestinal flora, such as immune tolerance to normal bacteria and immune rejection to pathogenic bacteria. Meanwhile, evidence from genetically engineered animal models suggests that nonpathogenic intestinal bacteria also play a critical role in the pathophysiology of UC 25. The gut microbial community in humans is equally significant, as it may be linked not only to disease pathogenesis but also to the degree of intestinal inflammation 26 and the type of disease, such as UC and Crohn's disease 27. Thus, UC is hypothesized to originate from the disruption of homeostasis between host mucosal immunity and the intestinal microbiota, resulting in an aberrant immune response of the organism to commensal non-pathogenic bacteria.

#### Dysregulated immune responses

It is well established that individuals with UC display aberrant innate and adaptive immune responses to microbial antigens. The inflammatory response is prolonged in UC by upregulating TNF and IL-1β expression, promoting neutrophil accumulation, and facilitating the formation of chromatin-based extracellular traps at sites of colonic mucosal damage 9. Antigens interact with macrophages and dendritic cells, thereby activating an intrinsic immune response. To enhance the recognition of microorganisms and antigens within the intestinal lumen, dendritic cells extend their dendrites through the intestinal epithelium 28. Numerous dendritic cells and macrophages in the lamina propria present antigens to B and T cells, thereby initiating adaptive immune responses. Activated and mature dendritic cells with stimulatory capacity are more abundant in UC patients, and their numbers increase with disease severity. This indicates that these cells play a key role in triggering and maintaining ulcers 29.

In UC, there is a shift in cytokine secretion that promotes inflammation and a subpopulation of dendritic cells with diminished tolerance emerges, exhibiting a lower capability to generate regulatory T cells 10. In general, effector helper T-cell (Th) responses and T-regulatory cell imbalances have led to dysregulation of several key cytokine production and shifted the focus of treatment for UC to anticytokine agents. These Th1/Th2/Th17-related cytokines, including TNF-α, interferon gamma (INF-γ), and series of interleukins, have been playing key roles in the pathophysiology of UC in various ways 30.

Toll-like receptors (TLRs) and NOD-like receptors are key microbial recognition receptors expressed by dendritic cells. The primary role of TLR signaling is to protect against infections, safeguard the epithelium from damage, and ensure the stability of both the intestinal microenvironment and the epithelial barrier. Specifically, TLR3 and TLR5 are predominantly expressed in normal intestinal epithelial cells, whereas TLR2 and TLR4 exhibit minimal or no detectable expression 31. TLR4 expression was significantly upregulated in UC patients' lamina propria cells 32.

### Barrier of ulcerative colitis

**Figure 3:**
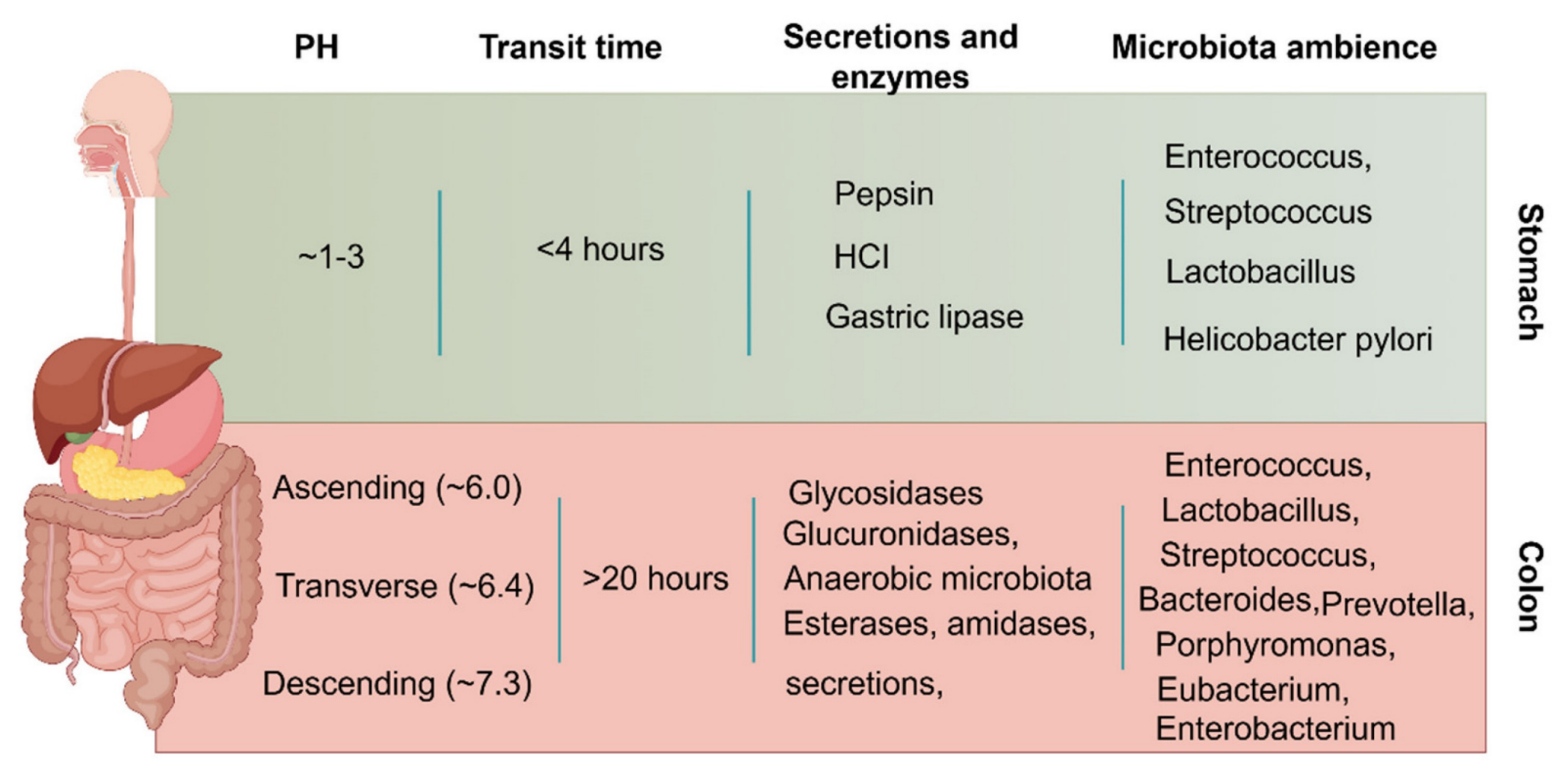
General physiologic considerations between stomach and colon faced by colon-targeted DDSs. Imaged by Figdraw.

Apart from the pathological factors, colonic administration depends on many physiological factors to ensure optimal efficacy after administration. The general physiological factors to be considered for colonic administration are given in (**Figure 3**).

Enzymatic activity, pH, motility, and fluid content vary significantly from the stomach to the intestine, and each organ has unique properties 33. In contrast to the stomach environment, the colon contains fewer active enzymes, lower levels of bile salts and digestive enzymes, lower penetration, gentler pH (6-8), lower fluid volume and active strength, and longer drug retention (up to 20 hours) 34. Drugs that are enzymatically degradable or poorly absorbed in acidic environments can therefore be sent to the colon, which is a perfect location for both of small and large molecule administration. Furthermore, the colon's abundance of lymphoid tissue allows for the quick generation of local antibodies that support successful immunization by absorbing antigens into the colonic mucosa's mast cells 17. In summary, although the colon is a somewhat good location for drug release and absorption, the microenvironment of the colon is very different in UC patients than in healthy individuals. This will greatly alter the efficiency of the drug reaching the lesion site and the subsequent therapeutic effect. Therefore, when designing UC-targeted colonic drug delivery, we must consider the pathological barrier characteristics of the UC state, and the following are some of the barrier factors that are often considered in the UC state.

#### GI tract pH

To ensure that drugs successfully reach the colonic lesion sites, it is essential to consider the marked pH variations all along the digestive system. The pH differences in the colon are a critical distinguishing factor between UC and a healthy colon. Specifically, the pH in an inflamed colon decreases from approximately 6.0-7.2 in a normal colon to 2.3-5.5 in the diseased state 35. Consequently, the pH gradient in the gastrointestinal tract (GIT) can be strategically used in the design of UC-targeted delivery systems to enable precise medicine administration to the lesion site. However, food consumption, microbial metabolism, and the active or remission status of the illness can all significantly alter the pH of the GIT in different people. This can impact the efficiency of the pH-dependent system and, in turn, the release of drugs from the region of interest 36. It is also worth noting that these pH changes may impact the colonic microbiome's composition, which will ultimately lead to the release and absorption of some drugs that are dependent on microbes or induced by enzymes secreted by microbes. This explains colonic targeting that relies only on pH causes inaccurate drug dosing, poor efficacy, and some toxicity associated with early/late drug release.

#### Gas

Besides liquids, gas pockets are encountered in the colon by pharmaceuticals. The colon typically contains 100 to 300 mL of gases, and the volume, location, and properties of these gases are greatly influenced by diet, transit time, and microbiota composition 37. The colon typically contains gases such as carbon dioxide, hydrogen, ammonia, nitrogen, methane, and sulfur-containing gases 38. With phosphorous dioxide levels of 11 mm Hg (~2%) in the ascending colon lumen and 3 mm Hg (~0.4%) in the sigmoid colon lumen, the colonic lumen is almost anaerobic. This causes in a negative oxygen gradient that rises at the rectum along the colon 39. While most colonic bacteria are specialist anaerobes, colonic epithelial cells adjust to hypoxic circumstances by changing the expression of certain genes 40.

According to recent research, H_2_S is linked to UC as well 41. H_2_S produced by specific gut bacteria can disrupt disulfide bonds in the mucus. Thus, one of the main mechanisms by which the mucus layer is disrupted is the dissolution of the polymeric MUC-2 network 42. The highest luminal concentration of H_2_S *in vivo* have been reported in the human colon (1.0-3.4 mM) 43. The rate of H_2_S production increases with UC severity. In UC patients, H_2_S concentrations are 2-3 times higher than in healthy individuals due to the increased number of sulfate-reducing bacteria 44.

#### Bacteria and enzyme

Over 400 distinct bacterial species can be found in the human colon (anaerobic and aerobic), and the enzymes present and chemical reactions occurring in the colonic area significantly influence the metabolism process of carbohydrates, fatty acids, proteins, and drugs 45. As UC progresses, the composition of bacteria and enzymes in the colon undergoes significant changes. For example, a decrease in *Bacteroidetes* and *Bifidobacteria* represent the start of UC evolution, while *Eubacterium* and *Peptostreptococcus* increase 36. Overexpression of some pro-inflammatory enzymes also exists in an inflammatory environment, including alkaline phosphatases (ALPs) 46, human neutrophil elastase (HNE) 47, and matrix metalloproteinases (MMPs) 48. In addition, when constructing UC-targeted therapeutic nanoplatforms, changes in enzyme activities in the gastrointestinal tract, including enzymes of pepsin, trypsin, lipases, as well as carbohydrate -degrading and bacteria-derived enzymes, must also be taken into consideration to ensure the stability of nanodrug delivery systems 49.

#### Mucus

The intestinal mucus, which overlays the intestinal epithelium and is associated with a monolayer of stem cells and columnar intestinal epithelial cells (IECs), is important in maintaining gut homeostasis. This layer is composed of several specialized cell types, including Goblet cells responsible for mucus secretion, Paneth cells involved in immunomodulation, Enterocytes for nutrient absorption, and Microfold cells (M cells) that facilitate antigen sampling. Together, these cells execute various physiological functions essential for tissue regeneration, immune regulation, and overall intestinal health. Physiologically, the mucus layer in the gut serves as a critical barrier to protect the host from exposure to external chemicals, such as drugs or toxins 50. Additionally, the mucus layer is essential for preserving electrolyte balance and promoting the colon's ability to absorb water. It serves as a barrier to shield the colonic epithelium from the abrasive impacts of fecal particles and successfully stops mechanical damage to the epithelium 51. Chyme can pass through the lumen thanks to the double layer of mucus covering the entire colonic epithelium, which also serves as a physical barrier between the microbiota and the colonic epithelium 52. While the inner mucus layer is firmly adhered to the epithelial cells, the outer layer, which consists of mucin and diluted antibacterial compounds, provides a typical habitat for commensal bacteria 53. Mucus covers the intestinal epithelium in the form of a gel, where water takes a high content consisted with inorganic salts, fatty acids carbohydrates, and glycoproteins 18. This mucus is constantly secreted by goblet cells to establish an effective protective barrier. Moreover, it promotes the clearance of foreign substances, such as drugs, upon contact, consequently minimizing the retention time of drugs within the mucus layer. Every 24 to 48 hours, the mucus layer completely regenerates due to the production of MUC-2 by goblet cells on the colonic epithelium. The old mucus is either spontaneously shed or broken down by the microbiota. Bacterial cells greater than 0.5 μm in diameter cannot pass through the interior mucus layer, which is linked to cup cells. The partial protease digestion has resulted in a looser structure in the exterior mucus layer, which is situated around 200 μm away from the epithelium 54. Due to the impacts of frequent mucus renewal and the mucus's physical barrier, this will inevitably have an impact on the effectiveness of drugs for UC.

#### Epithelial barrier

Villi are lacking from the colonic epithelium in contrast to the small intestine 55. Nonetheless, the apical surface of epithelial cells is characterized by the presence of microvilli. These microvilli, in conjunction with colonic crypts and unevenly folded mucosa, multiplied by 10 to 15 times the colon's surface area in comparison to a smooth tube 56. Colonic drug permeability is generally lower than that of the small intestine due to the smaller surface area. Nevertheless, most drugs classified as Class I in the Biopharmaceutical Classification System (BCS) are well absorbed, with a relative colonic bioavailability of over 70% 57. In contrast, BCS class III and IV drugs typically have lower colonic permeability and therefore bioavailability below 50% 58. The single monolayer of colonic epithelial cells is columnar in shape, and adjacent cells are joined by tight junctions 59. Given that tight junction proteins, which are intercellular adhesion molecules responsible for forming tight junctions, exhibit differential expression patterns in the descending and ascending colons, the descending colon demonstrates higher drug permeability 60.

The two main pathways for drug absorption through the intestinal epithelium are active transcellular transport and passive paracellular or transcellular diffusion. Hydrophilic drugs and those with large molecular weights are more likely to be absorbed through paracellular diffusion, which occurs at the tight junctions between adjacent epithelial cells 61. When intercellular tight connections are disrupted, for instance, epithelial integrity is jeopardized, paracellular drug diffusion may increase; this may occur in conditions such as UC, obesity, and type 1 diabetes 62. In contrast, transcellular diffusion through cells occurs in a positive correlation with the lipophilicity of the drug, as the drug must penetrate the epithelial cell lipid bilayer. The hydrophilicity/lipophilicity-influenced binding affinity determines the sensitivity of active drug transport across cell surface proteins. P-gp efflux transporter protein, for instance, has a higher affinity for cationic lipophilic substances 60. The drug's pKa and the pH of the gastrointestinal region are important determinants of its absorption pathway, as they will affect the degree of ionization of the drug and thus its hydrophilicity/lipophilicity.

## Classifications of Mesoporous Nanomaterial-Based Nanocarriers for Treatment of Ulcerative Colitis

Over the past few decades, MNMs have garnered significant attention for their biomedical applications, particularly as DDSs. Their unique mesoporous structure and high specific surface area offer numerous advantages over conventional drug nanocarriers. Firstly, MNMs have superior porosity and surface area enhance drug loading and release properties. Secondly, their particle and pore sizes can be precisely tailored through synthesis conditions to meet various drug delivery requirements. Thirdly, the surfaces of MNMs can be chemically modified to improve stability, selectivity, and targeting of drugs. Their structural integrity also prevents premature drug release *in vitro*, thereby facilitating more effective drug delivery. Finally, their high porosity enables a greater number of drug molecules to be loaded. Given these advantages, MNMs hold great promise for advancing DDSs design.

**Figure 4:**
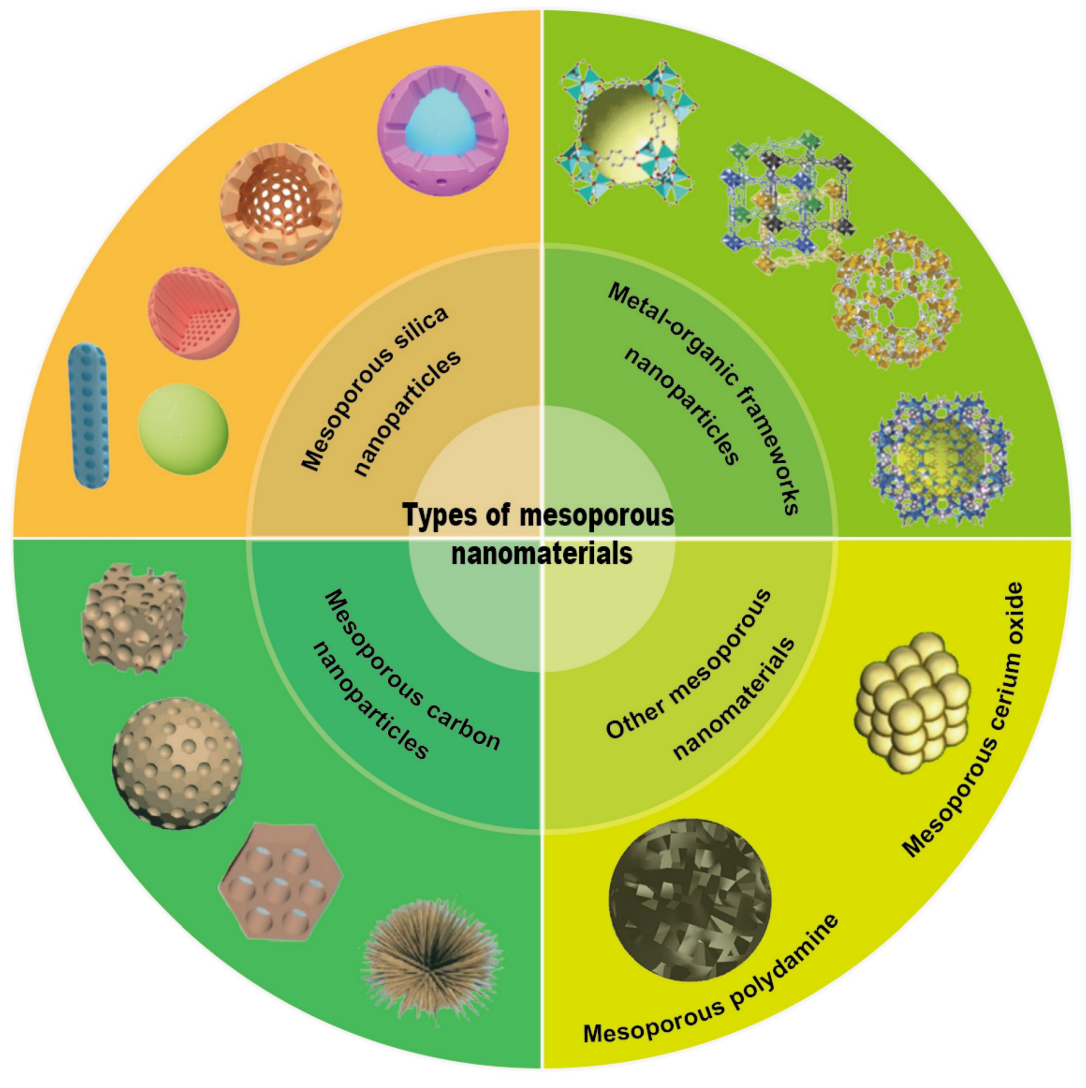
Different types of mesoporous nanomaterials.

As displayed in (**Figure 4**), the main MNMs that are often used for UC treatment include MSNs, MOFs, MPDA and other mesoporous nanomaterials. Combining the above advantages of MNMs, nanocarriers based on MNMs may modify the release rate and targeted distribution of medications to increase the therapeutic efficacy of UC and decrease pharmacological adverse effects. In the next section, we will discuss these MNMs and their applications in UC in detail.

### Mesoporous silica nanoparticles (MSNs)

Based on their compositional characteristics, MSNs can be classified into three categories: 1) pure silica MSNs, which are primarily composed of SiO_2_ and do not contain other elements or functional groups 63; 2) organic-inorganic hybrid MSNs, where organic functional groups are introduced into the silica framework 64; and 3) metal-doped MSNs, in which metals or metal oxides are incorporated into the silica matrix. Based on pore structure, MSNs are usually classified into various types, including hexagonal, non-porous, hollow, core-shell, and ultra-small or rod-shaped MSNs 65, 66.

As a typical MSNs, MCM41 was firstly synthesized with ordered two-dimensional (2D) hexagonal p6m arrangement of uniform mesopores 67. Another significant member of the M41S family is MCM-48, which has a three-dimensional (3D) pore structure that is bicontinuous and belongs to the cubic *Ia3d* space group 68. Another delivery carrier MSN that has been extensively studied in medicine is SBA-15, which has a 2D hexagonal *p6mm* structure 69. In addition, more complex geometries such as dendritic MSNs 70 or virus-like MSNs 71, 72 can be widely used in biomedicine.

**Figure 5:**
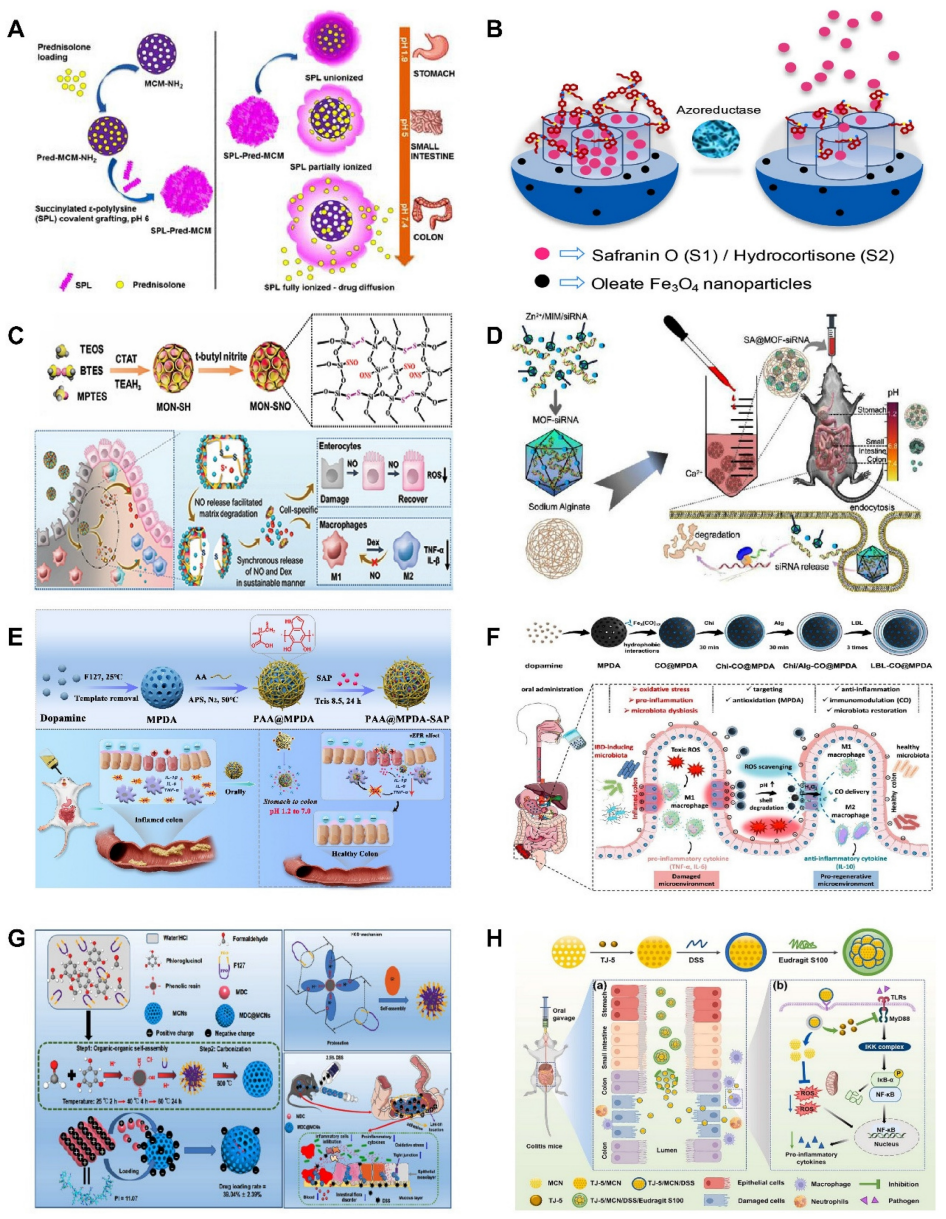
(A) Bifunctional SPL-coated MSNs for targeted colonic pH response and intracellular drug delivery. Reproduced with permission from 73, Copyright © 2017 American Chemical Society. (B) Smart gated magnetic MSNs for targeted colonic drug delivery. Reproduced with permission from 74, Copyright © 2018 Elsevier. (C) Schematic diagram for the preparation of the MON-SNO and MON-SNO@Dex performed therapeutic effects against DSS-induced acute colitis. Reproduced with permission from 75, Copyright © 2023 Junna Lu *et al.* (D) Schematic representation of SA@MOF-siRNA for the treatment of UC. Reproduced with permission from 76, Copyright © 2022 Meng Gao *et al.* (E) Design of the PAA@MPDA-SAP NPs for UC treatment. Reproduced with permission from 77, Copyright © 2023 Elsevier. (F) Schematic illustration of the preparation of LBL-CO@MPDA and its oral administration for targeting UC treatment via MPDA-mediated ROS scavenging and CO-induced immunomodulation. Reproduced with permission from 78, Copyright © 2023 American Chemical Society. (G) The construction principle of the MDC@MCNs nanoplatform and the accumulation of MDC@MCNs in the diseased colon to enhance the therapeutic efficacy of UC are systematically illustrated. Reproduced with permission from 79, Copyright © 2021 Ivyspring International Publisher. (H) Schematic illustration of the therapeutic effects of TJ-5/MCN/DSS/ Eudragit S100 in a UC model. Reproduced with permission from 80, Copyright © 2023 Elsevier.

In recent years, MSNs have been increasingly used as smart response nanocarriers in the treatment of UC and colon targeting. Pross Group 73 created a pH-responsive oral DDS based on MSNs functionalized with 3-aminopropyl groups that encapsulate succinylated ε-polylysine (SPL) to release the hydrophobic drug prednisolone preferentially in the colon, as shown in (**Figure 5A**), SPL-coated nanoparticles selectively released prednisolone in the colon pH condition (pH=5.5-7.4), but not in the more acidic environments of the stomach (pH=1.9) and small intestine (pH=5.0). This demonstrates the ability of SPL-coated nanoparticles to withstand the harsh conditions of the small intestine and stomach. In addition, formulations of micron-sized MSNs with magnetic nanoparticles were described by Teruel *et al.* 74 as potential techniques for targeted administration in the colon. Safranin O (S1) and hydrocortisone (S2) were loaded onto magnetic MSNs, and bulk azo derivatives with a urea component were used to functionalize the outer surfaces. At pH 7.4, an aqueous suspension of both solids exhibited very little payload release, but in the presence of sodium dithionite, considerable transport was seen as a result of azo bond breaking. Because of the longer retention time of the particles in the colon, the use of a magnetic belt improved the therapeutic efficacy of S2. As shown in (**Figure 5B**), prolonged retention time of magnetic nanoparticles in the colon enhances the therapeutic effect.

Similarly, polyethylenimine (PEI)-coupled diselenylated MSNs functioned as dual scavengers of ROS and cell-free DNA (cf-DNA) and inhibited pro-inflammatory macrophage activation by blocking cf-DNA-induced TLR9-MyD88-NFκB signaling, which effectively alleviated Dextran Sulfate Sodium (DSS) and 2,4,6-Trinitrobenzenesulfonic acid (TNBS)-induced colitis 81. Additionally, as demonstrated by Desai and colleagues, MSNs can be designed to target specific regions of the GIT 82. They assessed how impact on intestinal targeting of various arrangements of PEI-, polyethylene glycol (PEG-), and folic acid (FA)-coated MSNs (400-500 nm). The possible therapeutic advantages of MSNs loaded with the γ-secretase inhibitor (DAPT) for targeted intestinal epitheliudelivery to the m were then evaluated. Using oral gavage, mice were respectively administered PEG-, FA-PEI-, and FA-PEG-PEI-MSNs for three days in a row. As controls, unloaded nanoparticles and free DAPT were employed. When compared to the free drugs, the combination of MSNs loaded with FA-PEG-PEI DAPT produced a greater drug efficacy. They also assessed how surface modifications affected specific GIT regions. While the colon showed stronger affinity for PEG-PEI coatings. PEGylated nanoparticles have been shown to exhibit enhanced mucosal permeability, representing a practical modification strategy for colon-targeted drug delivery. This approach offers improved therapeutic options for colonic diseases, including ulcerative UC and colorectal cancer.

NO has shown promise as an immunomodulator for UC treatment. However, NO currently faces the problem of sudden explosive release and unsustainable release, which has led to consistently unsatisfactory therapeutic effects of NO. Therefore, developing a controlled NO-DDS with dual therapeutic role is necessary. Recently, Lu *et al.* 75 presented a hybrid mesoporous organosilica nanoparticles (MONs) with thiol-disulfide groups, where the NO-stored nanomaterials encapsulated the immunomodulator dexamethasone (Dex) after converting sulfhydryl groups to S-nitrosothiols (SNOs) through a nitrosation process, forming MON-SNO@Dex. As shown in (**Figure 5C**), the therapeutic window for NO-based colitis treatment is expanded by their enhanced NO storage and sustained release. The system can also release NO and Dex synchronously to synergize the treatment of UC, and its therapeutic effect is even more significant than that of 5-ASA, the first-line therapeutic drug commonly used in the clinic nowadays. In the treatment of UC and other inflammatory diseases, the potential therapeutic application of MON-SNO@Dex is demonstrated, providing valuable insights for the creation of safe and possibly successful treatment plans based on NO.

### Metal-organic frameworks (MOFs)

MOFs are well-defined porous inorganic-organic hybrid materials coordinated by metal ions and organic ligands 83. The metal ions act as ligand sites, while the organic ligands, typically characterized by rigid and spatially well-defined, form the backbone of the network structure 84. MOFs are materials that are crystalline, rigid, extremely stable, and highly porous. The ligand and associated structure can regulate the pore shape and crystal skeleton, which, owing to its remarkable stability, high drug-loading capacity, and pH responsiveness, has attracted considerable attention in the medical field 85, 86. There are four types of MOFs, including modified MOFs, pristine MOFs, MOF-derived materials, and MOFs consisting of natural enzymes.

MOFs can be further categorized into several types: (1) Isoreticular MOFs (IRMOFs) 87, (2) Zeolitic imidazolate frameworks (ZIFs) 88, (3) Porous coordination networks (PCNs) 89, (4) Materials institute lavoisier (MIL) MOFs 90, (5) Porous coordination polymers (PCPs) 91, (6) University of Oslo (UiO) MOFs 92, and (7) Cyclodextrin-based metal organic frameworks (CD-MOFs) 93. Instead of named by their coordination structures, there also emerged many MOFs named following the invented Universities, such as Hong Kong University of Science and Technology (HKUST-n) 94-99.

Small interfering RNAs (siRNAs) have been extensively explored as promising a pipeline of drug candidates for a range of inflammation-related diseases, given their superior safety and efficacy compared to antibody drugs. Despite progress, oral siRNAs formulations continue to be hindered by degradation in the GIT environment, limiting their ability to produce significant therapeutic effects in UC. Hence, Gao *et al.* 76 overcame the obstacle of siRNA penetration into inflammatory mucosal layers by encasing siRNA in MOFs. They created a hydrogel MOFs hybrid delivery method in which sodium alginate was used to encapsulate MOFs-loaded siRNA. To achieve the intended anti-inflammatory effect, the right-sized MOFs encouraged the mucosal layer of the colon to penetrate, and sodium alginate acted as a barrier to keep out stomach and intestinal fluids (**Fig 5D**).

Innovative approaches to formulating orally administered phytochemicals offer a promising avenue for addressing intestinal disorders. However, numerous challenges remain to be addressed, including low bioavailability, insufficient biocompatibility, and limited therapeutic efficacy. Chen *et al.* 100 used a crosslinked cyclodextrin metal-organic framework (CDF) encapsulated with resveratrol (Res) to produce Res-CDF, followed by its doping into hydrogel microspheres (Res-CDF in MPs) for targeted oral administration to alleviate UC. The MPs prevented Res from gastric acid, allowing it to reach the ulcerative colon site smoothly. In a mouse model, this system demonstrated effective anti-inflammatory effects, strengthening tight junction proteins to sustain the physiological function of the intestinal barrier. By leveraging the favorable properties of polysaccharide hydrogels and CDFs, this novel oral drug delivery system improves the bioavailability of phytochemicals. This enables the development of new oral interventions using natural phytochemicals for treating UC-related diseases and broadens the spectrum of phytochemicals available for therapeutic applications.

### Mesoporous polydopamine (MPDA)

Polydopamine (PDA), derived from marine mussels, exhibits significant potential for the early diagnosis and targeted drug delivery of diseases owing to its superior biocompatibility, biodegradability, and photothermal conversion properties. However, the application of conventional PDA nanoparticles is constrained by their limited drug loading capacity and encapsulation efficiency, particularly for hydrophobic drugs. The advent of mesoporous materials has expanded the possibilities in this field. Mesoporous polydopamine (MPDA) has been successfully applied in UC field due to its porous structure, simple preparation process, low cost, large specific surface area, high photothermal conversion efficiency, and excellent biocompatibility.

Wang *et al.* 101 constructed mesoporous polymers (MP), a ROS scavenging and gene interference therapeutic platform (MPDA-siRNA@CaP). MPDA NPs were used as porous carriers to carry TNF-α-siRNA and perform antioxidant functions, whereas PDA-induced calcium phosphate (CaP) coatings were used as pH-sensitive protective shells to prevent premature degradation of siRNA. Both *in vitro* and *in vivo* experiments demonstrated that system successfully alleviated DSS-induced UC, which integrated nanomedicine and gene therapy for a synergistic therapeutic effect.

Few monotherapies have demonstrated satisfactory long-term outcomes in the treatment of UC, prompting researchers to develop novel strategies that can effectively eliminate ROS while enhancing drug accumulation in the focal area, thereby improving the therapeutic efficacy for UC. Guan *et al.* 77 exploited the excellent drug-loading and free radical scavenging abilities of MPDA NPs with surface polymerization via polyacrylic acid (PAA). As shown in (**Fig 5**), the anti-inflammatory drug sulfasalazine pyridine (SAP) was successfully loaded into MPDA NPs to form nanomedicines (PAA@MPDA-SAP NPs). After oral intake, PAA could act as a "gate-keeper" that prevents the drug's burst release and breakdown in the stomach. Smoothly passing through the upper GIT, PAA@MPDA-SAP NPs can ultimately build up in the ulcerative colon. It effectively downregulates the expression of pro-inflammatory factors, strengthens the intestinal mucosal barrier, and ultimately alleviates colitis symptoms in mice through the synergistic integration of antioxidants and anti-inflammatory drugs. This lays the groundwork for future advances in UC nanotherapeutics. In addition to loading conventional drugs, MPDA can also be utilized to deliver specific anti-inflammatory gases for the treatment of UC. Zhang *et al.* 78 coated chitosan/alginate polyelectrolytes onto the surface of MPDA NPs loaded with carbon monoxide precursors (CO@MPDA) through a layer-by-layer (LBL) assembly approach. As shown in (**Fig 5**), benefiting from the pH-responsive properties of chitosan/alginate, the nanoparticles were able to reach the intestines smoothly. Owing to the negative charge on the LBL surface, electrostatic interactions enable the selective deposition of LBL-CO@MPDA onto ulcerative colonic lesions. By alleviating oxidative stress, LBL-CO@MPDA mitigates UC with restored immunological homeostasis, and the intestinal microbiota has also been modulated. This innovative nanotherapeutic strategy is demonstrated to have potential for targeted treatment of UC.

### Other mesoporous nanomaterials

In addition to the MNMs described above, various other MNMs are being used in the treatment of UC. For example, mesoporous carbon nanoparticles (MCNs) have also been explored for the UC. As shown in (**Fig 5**), Zhang *et al.* 79 developed a novel oral colon-specific drug delivery nanoplatform comprising *Musca domestica* cecropin (MDC) and MCNs. This colon-targeted delivery system may offer a more precise therapeutic strategy for UC patients, thereby enhancing the specificity and effectiveness of treatment.

Excessive ROS and a stressful inflammatory response are major characteristics of UC that can lead to disease progression and exacerbation. Liu *et al.* 80 developed a novel mesoporous cerium oxide nanoenzyme (MCN) capable of being loaded with the MyD88 inhibitor TJ-M2010-5. Colitis can therefore be synergistically alleviated by mitigating oxidative stress via ROS scavenging and suppressing the inflammatory response. As shown in (**Fig 5**), to enhance the targeting of this combination, the surface of the nano-enzyme was modified with DSS. Subsequently, Eudragit S100, an enteric coating material, was applied to the outer layer, thereby preventing premature release and absorption of the drug in GIT. The experimental results demonstrated that the nano-enzymatic system not only effectively removes excessive ROS but also simultaneously loads other drugs, thereby achieving dual functionalities of ROS scavenging and drug delivery.

## Strategies and Advances of MNMs-Based Smart Nanoplatforms for Targeting Nanotherapies against Ulcerative Colitis

The therapeutic agents for UC mainly include small molecule drugs, biological agents, gene drugs and nanozymes, etc. MNMs-based smart nanoplatforms for the nanotherapies of UC are categorized into intelligent drug delivery nanoplatform and drug-free nanoplatform according to their therapeutic payloads, each necessitating specific design strategies. Biological agents, such as monoclonal antibodies (e.g., anti-TNF-α, vedolizumab) and cytokines (e.g., IL-10), exploit MNMs' high loading capacity and protective coatings to enhance targeted delivery to the colon while preserving bioactivity. Gene therapies, including siRNA targeting pro-inflammatory genes (e.g., NF-κB, STAT3), utilize MNMs with endosomal escape ligands to facilitate efficient intracellular delivery. Small-molecule drugs, such as 5-ASA, corticosteroids, and targeted agents (JAK inhibitors), benefit from MNMs' tunable release kinetics, particularly redox-responsive systems that activate in the high- ROS microenvironment of inflamed colonic tissue. Combination therapies co-deliver biologics and small molecules (e.g., anti-TNF-α antibodies with 5-ASA) to synergistically suppress inflammation and promote mucosal healing. MNMs-based drug-free nanoplatform address UC pathology directly through ROS-scavenging cerium oxide nanoparticles or microbiome-modulating carriers that adsorb pathogens or deliver prebiotics.

**Figure 6:**
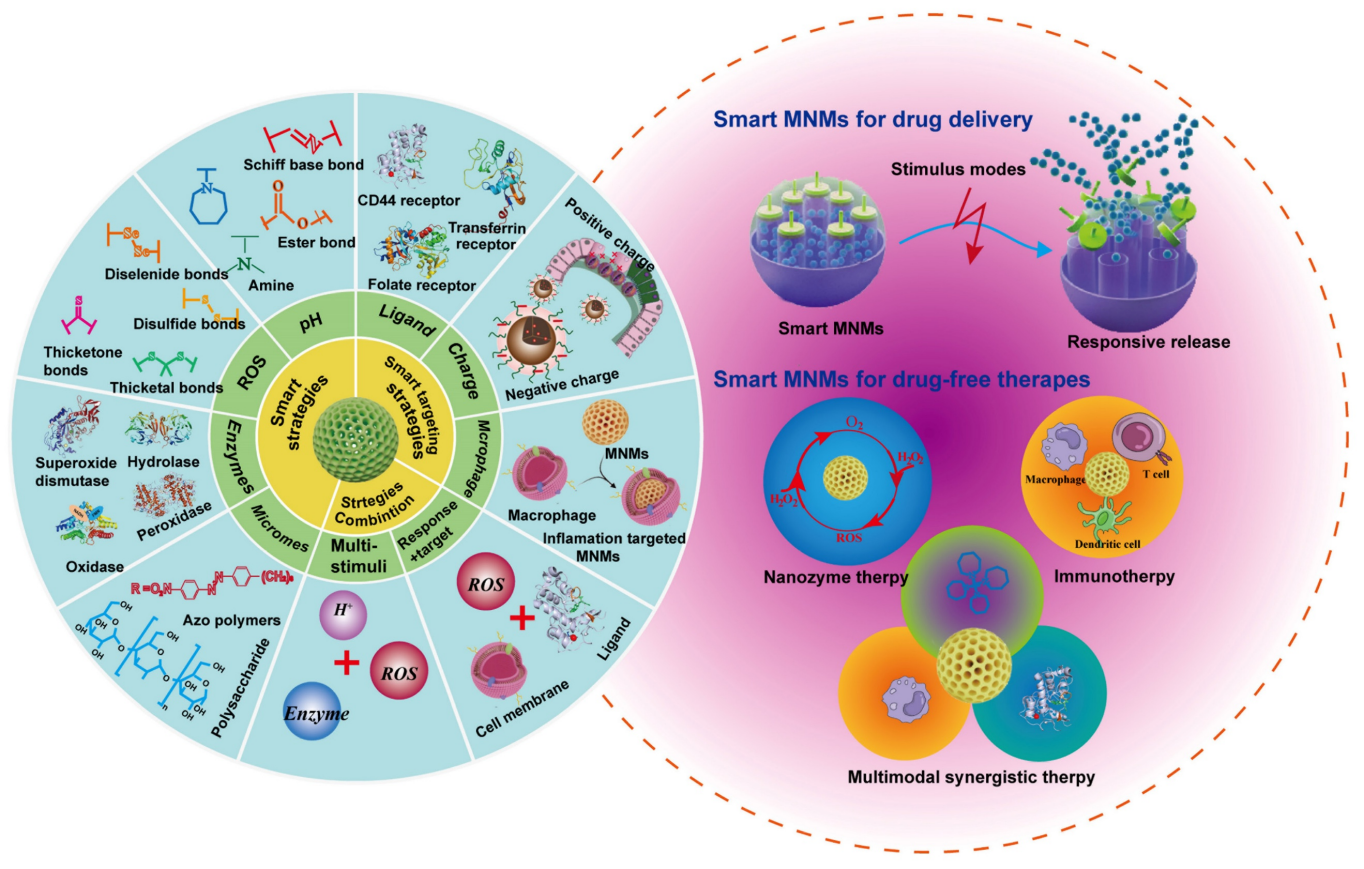
Various design strategies on smart nano-responsive platforms based on MNMs for UC applications.

The design of MNMs smart nanoplatforms should not only take into account physical therapy but also integrate the pathological features of UC. In UC, several microenvironmental alterations - including weakened intestinal epithelial barrier function due to the loss of tight junction proteins, increased permeability, decreased pH, increased release of inflammatory mediators, and accumulation of leukocytes and macrophages - characterize the pathological state. Given these features, researchers are increasingly inclined to combine MNMs with pathological characteristics to develop various smart nanoplatforms for targeted delivery of therapeutic agents specifically to UC sites. (**Figure 6**) illustrates the design strategies for these smart nanoplatforms based on MNMs.

### Strategies of MNMs-based smart nanoplatforms for overcoming oral therapeutic barriers against ulcerative colitis

As is well known, overcoming the GIT physiological barriers are the key to ensure colon-targeting of oral therapies, where the pH conditions, enzyme content, mucus status, transit time, and the microbiota diversity also make a difference. Here, we focus on various strategies of MNMs smart nano-responsive platforms to cross the pH and mucus barriers.

Coating MNMs with pH-sensitive biocompatible polymers represents an effective strategy for addressing pH-related challenges in the GIT during colonic drug delivery. To enhance therapeutic efficacy, this coating technology not only facilitates controlled release within a specific pH range but also protects the encapsulated active pharmaceutical ingredients from adverse GIT conditions, such as gastric acid, bile acids, and microbial degradation. Pross Group 73 selected SPL as a coating material for MSNs, which is predicted to prevent drug leakage in gastric pH environment and facilitate drug release through pH-dependent conformational changes in intestinal pH. It was found that this smart nano drug delivery system for MNMs can control drug release of hydrophobic drug prednisolone from MSNs under different pH conditions in the GI tract.

Controlled release throughout GIT is difficult because of the rapid secretion and shedding of gastrointestinal mucus, which effectively traps and quickly removes many types of nanoparticles. Therefore, to prolong the residence time of MNMs in the mucus layer, some strategies that promote mucus adhesion or penetration are often employed to prolong the retention time of MNNs nanoparticles within the mucosal layer, thereby potentially improving oral drug delivery. Inspired by the probiotic *Escherichia coli* strain Nissle 1917, Xu *et al.* 102 coated the cell membrane on the surface of MSNs to construct a nano-drug delivery system (SeM@EM). The cell membrane-based nano-delivery platform faithfully replicated the specific characteristics of the source cells, thus making MSNs strongly adhere to the mucus layer, reducing the amount of drug removed by mucus, and enhancing the efficacy of drugs on UC. Currently, smart nano delivery platforms capable of penetrating mucus barriers can also be engineered 103, 104.

### Strategies of MNMs-based smart nanoplatforms for targeting drug nanotherapies against ulcerative colitis

UC is a chronic inflammatory disease which shows persistent inflammation of the intestinal mucosa. Traditional therapeutic approaches, which often involve anti-inflammatory drugs and immunosuppressive agents, frequently face challenges due to significant side effects and limited efficacy. MNMs-based smart nanoplatforms facilitate precise regulation of drug release and improve targeting specificity. They are designed to respond to specific stimuli-such as pH, enzymes, ROS, microbes, and receptor interactions, facilitating the controlled release of therapeutics at the site of inflammation. This targeted approach aims to maximize therapeutic efficacy while minimizing systemic side effects, thereby offering a promising avenue for UC treatment.

In the next section, we will introduce various smart nano-responsive platforms based on MNMs for the treatment of UC, which take advantage of the various pathological features of UC (significant decrease in pH, abnormally high ROS concentration, etc.) or even a combination of several UC pathological features to achieve 'targeted and quantitative' release of drugs.

#### ROS responsive MNMs-based smart nanoplatforms

Low levels of ROS, which act as inflammatory mediators, are generated in normal tissues, but they are significantly overexpressed at sites of inflammation 105. Oxidative stress resulting from overexpression of ROS is a basic feature of inflammatory diseases 106. Furthermore, the level of oxidative stress is positively correlated with the severity of inflammatory diseases 107. It is already well known that the imbalance between ROS production and antioxidant capacity in diseased intestinal tissues plays a key role in the pathophysiology of UC. According to reports, intestinal mucosal ROS concentrations in UC patients are 10-100 folds greater than in healthy individuals. These concentrations are limited to the disease site and cause oxidative tissue damage, which exacerbates the start and progression of UC 108, 109. According to clinical studies, UC patients exhibit consistently reduced antioxidant capacities even during remission, leading to oxidative stress-induced damage. Several reports have documented significantly lower levels of SOD and glutathione peroxidase (GPx) activity in the colonic mucosa of UC patients 110.

**Figure 7:**
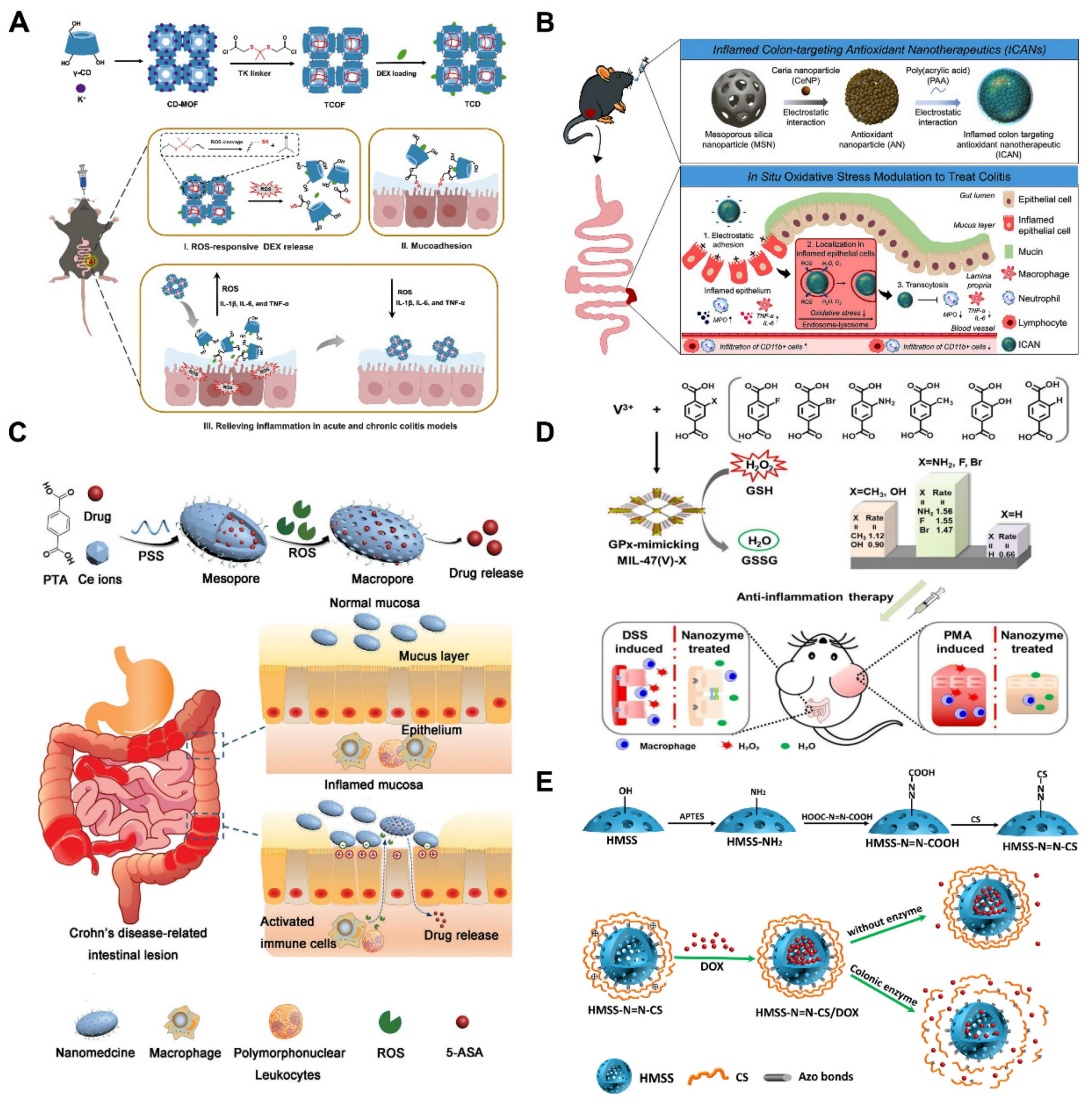
(A) Schematic diagram of the fabrication of TCD and the process thereof in combination with drug-responsive ROS release *in vivo* to treat UC. Reproduced with permission from 111, Copyright © 2024 WILEY. (B) Schematic representation of the construction of ICANs and the proposed mechanism of oxidative stress regulation in UC. Reproduced with permission from 4, Copyright © 2023 American Chemical Society. (C) Synthesis of 5-ASA@Ce-MOF@PSS and drug release at inflammatory intestine. Reproduced with permission from 112, Copyright © 2020 WILEY. (D) Schematic diagram of the synthesis of rationally designed GPx-mimicking MIL-47(V)-X MOF nanozymes for anti-inflammation therapy. Reproduced with permission from 113, Copyright © 2021 WILEY. (E) Construction of MSNs nanoplatform based on colonic enzyme response and its responsive drug release process. Reproduced with permission from 114, Copyright © 2020 Defu Cai *et al.*

Considering the critical role of ROS levels in UC treatment, ROS-sensitized MNMs with inflammation-targeting function are more suitable for enhancing drug efficacy than conventional nanoparticles. Therefore, Xiong *et al.* 111 proposed covalently cross-linked frameworks containing ROS-responsive thioketal (TK) (termed TCOF) as triggerable carriers, and also, to illustrate the advantages of TCOF in targeting therapeutic treatment and modulating drug release, non-ROS-cleavable alkyl linker (pimeloyl chloride) were used in place of TK linker in TCOF, and the TK linker in TCOF were synthesized with physicochemical equivalent properties of non-ROS-responsive covalent cross-linked frameworks (CCOF). To illustrate the benefits of precision medicine for the treatment of UC, DEX loaded in TCOF (TCD) and CCOF (CCD) were studied. TCD selectively activated the release of DEX following the collapse of nanoparticles caused by ROS. Furthermore, TCD decreased inflammation levels *in vitro* and successfully shielded cells from oxidative stress damage. Notably, the TK moiety of TCD is oxidatively destroyed by consuming ROS at the site of inflammation, exposing the thiol moiety to mucosal adhesion, thereby enhancing the retention of TCD in the colon. *In vivo* studies of UC treatment have demonstrated that oral TCD exhibits superior inflammation-targeting properties, significantly attenuates oxidative stress and ameliorates acute and chronic colitis compared to CCD. Polymer particles with a robust backbone structure can be fabricated by incorporating cross-linking agents to couple the hydroxyl groups on the cyclodextrin (CD) units in CD-MOF, ensuring stability after oral administration. In addition, in mice, TCD demonstrated an outstanding safety profile. These results are highly promising for advancing precision therapy and personalized medication delivery for UC (**Figure 7A**).

To effectively treat UC, there is an urgent need for safe, orally administered targeted therapies. Min *et al.* 4 loaded ceria nanoparticles (CeNPs) onto porous MSNs, where poly (acrylic acid) (PAA) was also coated on the surface to expose a negative surface charge, thereby developing inflamed colon-targeted nanotherapeutics (ICANs). By utilizing the pH-responsive characteristics of the PAA coating, this system enables both colonic localization and mucosal adhesion. As illustrated in (**Figure 7B**). ICANs regulate the amount of oxidative stress in inflammation by preferentially adhering to the inflammatory epithelium and spreading throughout the ulcerative colonic tissue. The management of redox balance leads to a decrease in immunological responses and infiltration of inflammatory cells, and an increase in the regeneration of epithelial barrier function. These results emphasize potentially noninvasive ICANs may be an effective strategy for treating UC by reducing oxidative stress levels in colitis.

Yin *et al.* 112 reported an oxidation-responsive metal-organic framework material (Ce-MOF@PSS). Dependent on the surface modification, the obtained Ce-MOF@PSS with negative surface charge can preferentially adhere to the inflamed colon site (**Figure 7C**), and then, due to the overexpression of ROS at the site of inflammation, the trivalent cerium ions were converted to the tetravalent cerium ions, which induced changes in the pore size of the MOFs to be realized, and the drug was subsequently released from it. Although MOFs have been extensively studied, such MOFs with pore-size transforming ability have been less frequently investigated. GPx played an important role in maintaining the balance of ROS *in vivo*. Wu *et al.* 113 skillfully utilized MOFs nano-enzymes to mimic the effect of GPx by linking different substituted ligands through a ligand engineering strategy. As illustrated in (**Figure 7D**). The *ex vivo* and *in vivo* experiments showed good antioxidant effects and effective alleviation of UC, expanding the application and research of MOFs in bio-nanomedicine.

#### Enzyme-responsive nanomaterial-based smart nanoplatforms

Under mild cell pressure, pH, and temperature conditions, enzymes operate as catalysts to carry out chemical processes at remarkably rapid rates 115. Compared to normal tissues and organs, inflammatory environments have different enzyme activities. Macrophages migrate to the site of injury during an immune response, and inflammatory cells release a variety of proteins and enzymes related to inflammation, including lysozyme, MMPs, hydrolase, myeloperoxidase, cytokines, and chemokines 116. Moreover, UC is associated with overexpression of certain inflammatory proteins generated by inflammatory signaling pathways, such as cyclooxygenase-2 (COX-2) 117. Thus, these enzymatic cues could serve as targets for drug delivery in UC, triggering drug release at sites of inflammation. Cai and co-workers. 114 prepared biodegradable chitosan (CS) via azobonds (HMSS-N=N-CS) which is cleavable and enzyme-responsive for colon-targeting delivery. As illustrated in (**Figure 7E**), for the purpose to encapsulate doxorubicin (DOX), they constructed hollow mesoporous silica spheres (HMSS), and external CS attached to the surface of the HMSS via azobonds thus blocking the mesopores of HMSS. This set up effectively protected the integrity of the DOX in the upper part of the GIT. The azobonds between the HMSS and the CS are enzyme cleavable at the colonic site, leading to the release of DOX. This work pioneered the MSNs-based colon-targeted drug delivery and also established the basis for developing an enzyme-responsive, UC-targeted nanoplatform based on MNMs.

#### Microbially responsive nanomaterial**-**based smart nanoplatforms

It has been established since the Human Microbiome Project concluded in 2012 that symbiotic bacteria have a significant impact on health and disease and encode 150 times more unique genes than their human hosts 118, 119. The human microbiota consists of approximately 100 trillion microbial cells, with the majority concentrated in the GIT, particularly in the colon, which exhibits the highest bacterial density (10^12^ cells per gram of fecal content) 120. These bacteria have an amazing ability to metabolize drugs and facilitate their transport. For instance, colonic bacteria could activate prodrugs. The earliest prodrug that depends on intestinal bacteria to activate its active ingredient, 5-ASA, is sulfasalazine 121. Systems that depend on the microbiota have been thoroughly studied to deliver drugs to the colon precisely 122. Certain polymers serve as attractive coverings for colonic release dosage forms because they are selectively absorbed by colonic bacteria despite being indigestible in the proximal gut 123.

**Figure 8:**
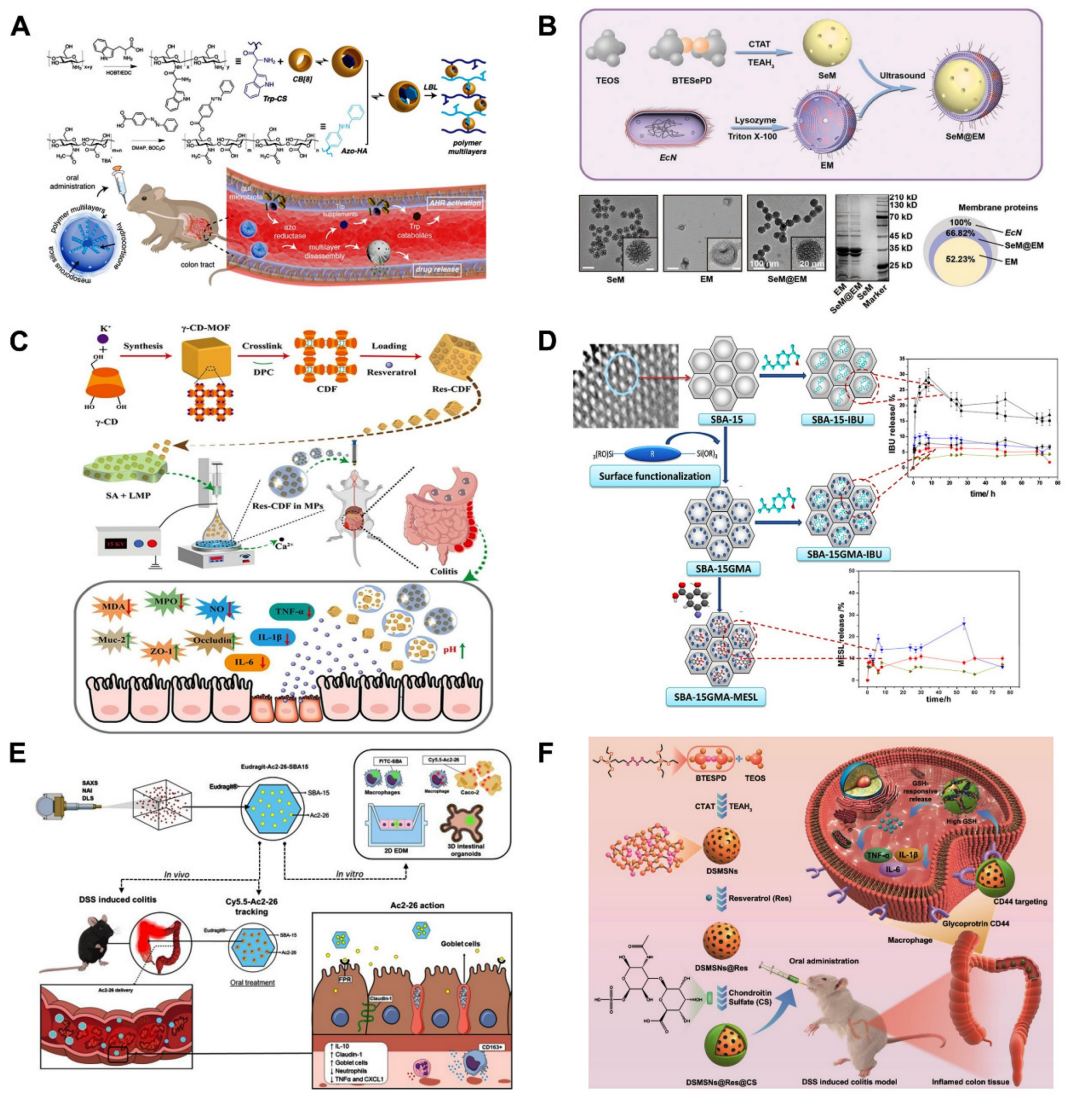
(A) Schematic illustration of fabrication and mechanism of MSNs for colon-targeted drug delivery. Reproduced with permission from 129. (B) Schematic illustration of the synthesis and characterization of SeM@EM. Reproduced with permission from 102, Copyright © 2022 WILEY. Copyright © 2022 Elsevier. (C) Schematic illustration of the synthesis of Res-MOF in MPs as oral drugs for colitis. Reproduced with permission from 100, Copyright © 2023 Elsevier. (D) SBA modified for colon-targeted delivery of mesalamine scheme diagram. Reproduced with permission from 130, Copyright © 2015 Elsevier. (E) Schematic diagram of EL30-D55-coated SBA-15 containing Ac2-26 for the treatment of UC. Reproduced with permission from 131, Copyright © 2024 Broering MF *et al.* (F) Schematic illustration of the DSMSNs@Res@CS preparation procedure and UC targeting treatment. Reproduced with permission from 132, Copyright © 2023 Elsevier.

Using microbial responsive triggering to achieve UC-targeted drug delivery is considered the most effective strategy 124. In the microbiota-triggered delivery system, the cargo is released in the colonic region after the encapsulated/bound polysaccharides undergo enzymatic degradation by gut enzymes and are primarily metabolized by colonic bacteria 125. Polysaccharides and azo polymers are the two primary kinds of polymers that have been developed to date 126. Because azo polymers may cause cancer and require the use of organic solvents during manufacture, their continuous use in humans has been discontinued 127. Therefore, polysaccharide-based formulations are key facilitators for colonic drug release enzyme-sensitized systems. In recent years, tryptophan (Trp) metabolites produced by gut microbes have played an important role in the pathogenesis and treatment of UC and balancing immunological reaction of the mucosa by binding to the aryl hydrocarbon receptor (AHR). The literature suggested that Trp supplementation was an optimized approach for the prevention and treatment of UC 128. Cheng and colleagues. 129 developed multilayer-coated mesoporous silica (MSs), which were capable of specifically releasing loaded drugs in the colonic region under conditions of azoreductase production by intestinal microbes, the system modified chitosan (CHI) with Trp and hyaluronic acid (HA) with azobenzene (Azo), respectively, and introduced the host-guest recognition between cucurbit[8]uril (CB[8]) and Trp/Azo in order to prepare the CHI/HA/MSN system for the treatment of UC (**Figure 8A**). More importantly, restoring tryptophan metabolism by the microbiota induced activation of the AHR, which in turn increases anti-inflammatory effects. Notably, although MSs remained stable at both acidic and neutral pH, they showed excellent responsiveness to dithionite, an azo reductant used to mimic the environment of specific azoreductases *in vitro*. Mice with increased levels of AHR agonists, regulation of inflammatory cytokine production, and improved colonic epithelial barrier integrity showed improved therapeutic benefits of MSs in DSS-induced UC. This innovative delivery strategy is anticipated to have significant promise for the future management of UC.

Cell membrane-based nanomedicines, replicating cell-specific properties intact, are intriguing therapeutic platforms that combine the functional diversity of nanomaterials with the biomimetic qualities of cell membranes to treat UC. In view of this, Xu *et al.* 102 used *Escherichia coli* strain Nissle 1917-(*EcN*) membrane (EM) coated with diselenide-bridged MSNs (SeM) to establish mesoporous nanosystems responsive to microbial organisms (SeM@EM) in order to facilitate the comprehensive treatment of UC by maintaining redox and immune responses in the gut to achieve homeostasis and modulating the function and flora diversity of the intestinal microbiome (**Figure 8B**). In a mouse model of acute colitis brought on by DSS, SeM@EM recovered intestinal redox balance and immunomodulatory homeostasis after oral treatment by adhering firmly to the mucus layer. Strong antioxidative and anti-inflammatory qualities are also present in SeM, a bioactive and biocompatible nanomaterial that can scavenge ROS. This microbial membrane-based treatment for UC, combining the biomimetic properties of cell membranes with the superior characteristics of mesoporous materials, will show promise as a treatment platform for inflammatory disorders, including UC.

#### pH-responsive mesoporous nanomaterial-based smart nanoplatforms

The body regulates a variety of factors, including pH, temperature, and glucose content, *in vivo*. Phosphate buffering keeps the pH of our body's normal tissues and blood approximately at 7.4 133. Each organ and tissue have a pH that is ideal for its particular function and specificity. For instance, the small intestine and colon have slightly lower pH than normal tissues, 5.5-6.8 and 6.4-7.0, respectively 134, while the stomach has a moderately acidic pH of roughly 1.5-3.5. Furthermore, the inflammatory environment typically exhibits a slightly acidic condition at pH 6.5 116, making the use of pH-responsive strategies for UC treatment highly promising.

As displayed in (**Figure 8C**), Chen *et al.* 100 constructed Res-CDF by encapsulating Res in a crosslinked CDF. This was then doped into naturally occurring polysaccharide hydrogel microspheres (Res-CDF in MPs), and the design was able to specifically release Res-CDF in response to the mildly alkaline environment of the intestinal tract, leading to sustained release of Res. By increasing the bioavailability of phytochemicals through the use of polysaccharide hydrogels and CDFs, this unique oral delivery strategy lays the foundation for the development of novel oral therapies that use natural phytochemicals to treat UC. Rehman *et al.* 130 synthesized hybrid SBA-15GMA with glycidyl methacrylate (GMA) bridging chain as a carrier for mesalamine (MESL) (**Figure 8D**), they investigated the drug-carrying effect of mesoporous silica SBA-15 and its hybrid SBA-15GMA as mesalamine carriers. The results showed that SBA-15, after functional group modification, has good colon-targeting function. *In vitro* release studies have shown that modified silica SBA-15GMA is able to preferentially release MESL at high pH and can be considered for colon-targeted delivery systems. This provides new modification strategies for the development of MNMs for UC treatment, facilitating the use of more effective MNMs.

Steroids are also often used in the treatment of acute UC. However, the low delivery efficiency and side effects of these drugs in conventional dosage forms limit their widespread use. Qu *et al.* 135 synthesized sensitive to pH to treat UC, Eudragit-mesoporous silica nanocomposites will allow glucocorticosteroids administration in the colon, taking advantage of the fact that the Eudragit material does not degrade under gastric acidity and the large loading capacity of MSNs, which is easy to be functionalized on the surface, to achieve UC target. The specific release aims to avoid systemic absorption of steroids and reduce side effects, broadening the path of steroid treatment for UC. AnxA1 is a 37 kDa endogenous protein comprising the calcium and phospholipid-binding membrane-bound protein superfamily. It is a key endogenous anti-inflammatory and tissue repair protein in UC. Broering *et al.* 131 developed MSNs loaded with Ac2-26 and capped with Eudragit^®^ L30-D55 for the treatment of UC via the oral route. As illustrated in (**Figure 8E**), this nanoplatform protects biologic therapeutic drugs, such as peptides, from the adverse effects of the gastrointestinal tract and prevent their premature release, providing a simple and highly effective non-invasive way to treat UC.

#### Macrophages responsive nanomaterial**-**based smart nanoplatforms

It has been established that in UC, ROS-induced immune dysregulation significantly polarizes macrophages toward a pro-inflammatory phenotype (M1) 136. The activation of M1 macrophages induces the secretion of various cytokines, including IL-6, IL-1β, and TNF-α, thereby sustaining a pro-inflammatory microenvironment within the colon 137. Certain receptors, including phosphatidylserine receptors, CD44, and folate receptors (FRs), are markedly upregulated on the surface of macrophages as inflammation progresses, enabling precise targeting inflammatory sites. As a result, macrophage-targeting strategies offer effective treatments for UC. HA, a major component of the extracellular matrix in human tissues, exhibits a high affinity for CD44, a receptor predominantly expressed on macrophages. Consequently, HA can serve as an effective macrophage-targeting ligand, enabling precise and targeted drug delivery to macrophage-rich inflammatory sites. Zhao *et al.* 138 successfully constructed a CD-MOFs-based oral sequence-targeted delivery nanoplatform, namely QT-CMOF@HA. To precisely deliver quercetin (Qu) to inflammatory colonic lesions, a new oral delivery platform called QT-CMOF@HA nanocubes based on hydrophobic cross-linked CD-MOF (CMOF) is presented. First, a mitochondrially functionalized Qu-(5-carboxypentyl)-TPP precursor complex (QT) was loaded into CMOF to enhance mitochondrial targeting. Then, CMOF was coated with a chitosan/glutathione-responsive HA shell to form the QT-CMOF@HA nanoplatform, improving gastrointestinal tolerance. The results demonstrated that QT-CMOF@HA significantly reduced the production of inflammatory cytokines by promoting the macrophage repolarization from M1 (pro-inflammatory) toward M2 (the anti-inflammatory) phenotype. Additionally, it improved gut microbiota composition and enhanced intestinal mucosal barrier integrity. QT-CMOF@HA nanocubes present great potential for UC treatment, providing a multimodal strategy to target inflammation and promote mucosal healing in a controlled and targeted manner.

The HA coating promotes binding to the CD44 receptor; however, it rapidly degrades in the stomach, diminishing its ability to target colonic lesions. To protect the encapsulated drug from degradation in the acidic environment, the outer layer material of MNMs must possess resistance to degradation by gastric acid. Lei *et al.* 139 effectively filled Cur in UiO-66, where the amino-functional groups on the surface and following grafted layer of HA. The PDA layer was subsequently employed to form colon-targeted Cur@MOF@HA-PDA NPs for treatment of UC. Cur@MOF@HA-PDA nanoparticles can enhance the stability of Cur, and possess acid resistance and ROS responsiveness, enabling effective delivered to the UC lesion site, where Cur is released for enhancing therapeutic efficacy after oral administration. According to *in vitro* research, the Cur@MOF@HA-PDA nanoparticles could boost M2 polarization, suppress M1 polarization of macrophages, and remove intracellular ROS. Furthermore, *in vivo* studies shown the administrated Cur@MOF@HA-PDA NPs might successfully reduce intestinal inflammation symptoms and promote intestinal tissue regeneration in UC animal models. Moreover, intestinal immune function could be modulated to suppress inflammatory responses and enhance M2 macrophage polarization. In summary, the Cur@MOF@HA-PDA NPs, exhibit remarkable efficacy in the treatment of UC, as colon-targeted drug delivery nanoplatforms.

#### Multi-responsive mesoporous nanomaterial-based on smart nanoplatforms

Although the smart-responsive nanoplatforms based on MNMs have achieved some success in UC treatment, a single-response strategy is insufficient to ensure that the drug reaches the lesion site smoothly. This often leads to premature or delayed drug release, which can hinder UC treatment and may also cause unexpected toxic side effects. Considering that, multiple smart response nanoplatforms based on MNMs have emerged. Jiang *et al.* 132 prepared an oral drug delivery vehicle of organosilica nanoparticles (DSMSNs) containing tetrasulphide, surface-coated with CS to achieve targeting (**Figure 8F**). CS can specifically bind to the transmembrane glycoprotein CD44, which is overexpressed by macrophages in the UC environment, resulting in the selective accumulation of macrophages and colon epithelial cells, and also, as in the UC state, GSH levels increase and the tetrasulphide combination is disconnected by GSH, enabling DSMSNs to release Res, this multiple response and targeted smart delivery strategy will be a promising platform for UC therapy.

Nowadays, it has been increasingly recognized that H_2_S is also closely related to the development of UC. To address this, Dou *et al.* 92 prepared octahedral UiO-66 as an adsorbent and scavenger of H_2_S, modified UiO-66 with Cux-RhodamineB (CR), and then dispersed it in hydroxypropyl methylcellulose acetate succinate (HPMCAS) solution, formulating HF@UiO-CR (HUR) microfluidic droplets by microfluidic technique. The system exploited the pH sensitivity of HPMCAS, which allowed the cargo to successfully reach the intestine, and subsequently, then, by electrostatic interactions, the negatively charged UR attached itself to the inflammatory colon, and the UR adsorbed excess H_2_S, which, along with the reduction of H_2_S, preserved epithelial cell integrity and promoted the formation of tight junction proteins, and also increased the abundance of gut microorganisms, in a DSS-induced mice model of UC that HUR is expected to be a promising therapeutic platform for UC treatment. *In vivo* testing of MSNs (S2) capped with hydrolyzed starch in rats was studied by González-Alvarez *et al.* 140. The study employed Eudragit® FS 30D enteric-coated gelatin capsules containing S2. The findings demonstrated that the encapsulation of safranin O within the S2-Eudragit® FS 30D formulation led to selective accumulation of the dye in colonic tissue. This targeted biodistribution profile is highly advantageous, suggesting that this formulation, or analogous systems utilizing gated mesoporous silica nanoparticles (MSNs), could serve as a promising platform for UC-targeted drug delivery. Such an approach may enhance therapeutic efficacy while minimizing systemic side effects. The integration of conventional pH-sensitive responsive materials with mesoporous silica nanoparticles (MSNs) to fully leverage their individual characteristics and achieve a synergistic effect may significantly enhance the efficacy of future treatments for UC.

### Strategies of MNMs-based smart nanoplatforms for targeting drug-free nanotherapies against ulcerative colitis

#### Macrophage-regulating therapy

As mentioned above, macrophages are a critical component of the innate immune response and play a crucial role in maintaining the homeostasis of intestinal microenvironmental. UC is a chronic inflammatory disease that is closely associated with immunological dysfunction. Increasing evidence suggests that macrophages are promising non-pharmacological targets of action for regulating the inflammatory microenvironment and the gut immune system 141, 142, when UC occurs, several signaling pathways (e.g. NF-κB 143, MAPK 144, PI3K/Akt 145, Wnt/β-catenin 146, and AMPK 147) are engaged in controlling the macrophages' release of inflammatory mediators and inflammatory factors; these pathways interact and control the inflammatory microenvironment of UC. Meanwhile, receptors that are highly expressed on macrophages during UC progression, such as folate receptor 148, CD44 149, CD98 150, peptide transporter protein 1 (PepT1) 151, mannose receptor 152, and Dectin-1 153, provide potential binding sites for active drug-targeted response delivery. A few researchers have leveraged this property in conjunction with the significant advantages of nanotechnology platforms, committing extensively to the field and achieving notable results 154, 155.

**Figure 9:**
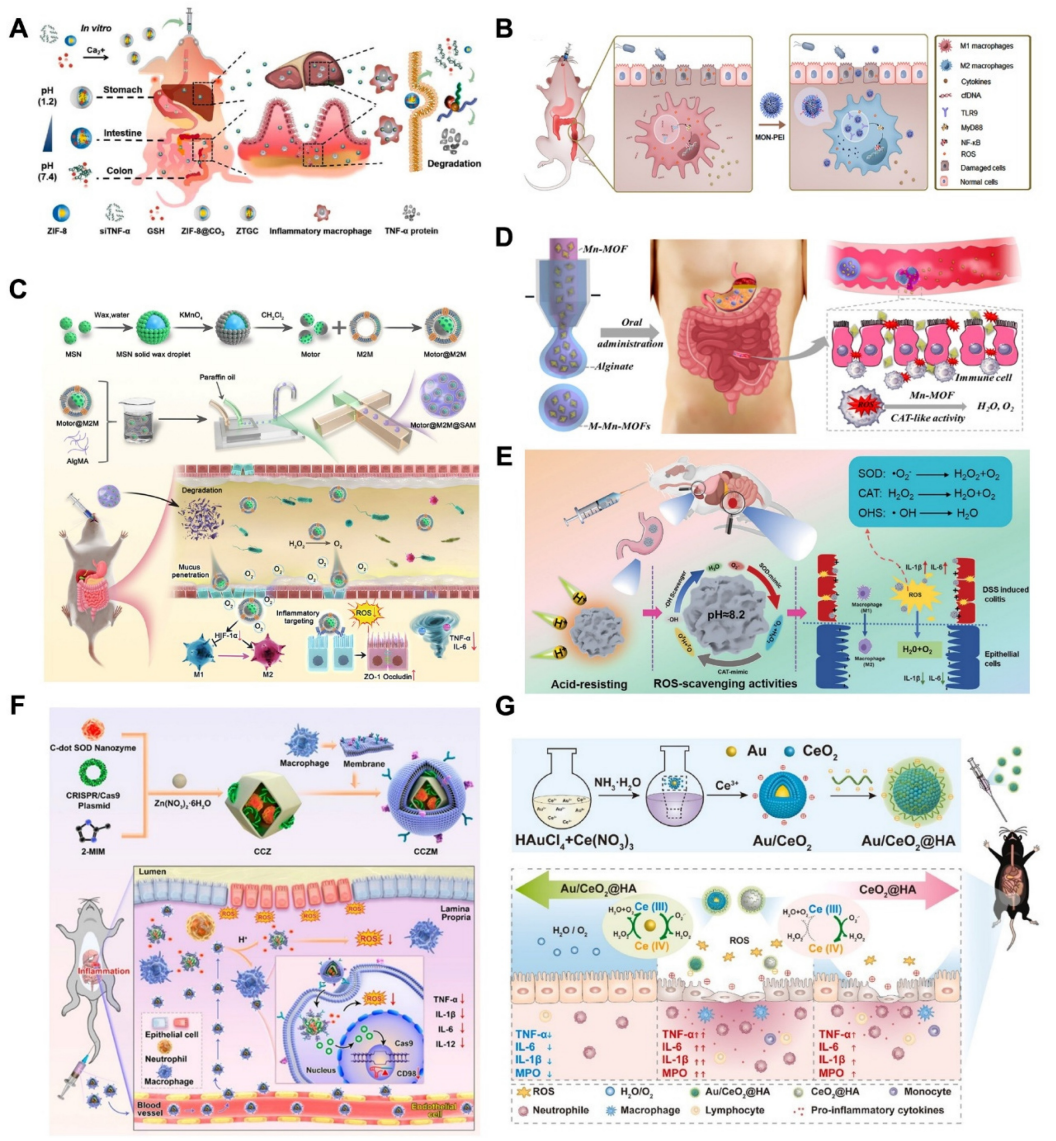
(A) Schematic illustration of biomimetic mineralized MOFs reprogrammed inflammatory macrophages for the treatment of UC. Reproduced with permission from 157, Copyright © 2023 Elsevier. (B) Schematic of the design of a biodegradable nanomedicine with cfDNA- and ROS-scavenging activity for UC therapy. Reproduced with permission from 81, Copyright © 2022 Chengxin Shi *et al.* (C) Schematic diagram of Motor@M2M@SAM formulation and its action for treatment of UC. Reproduced with permission from 158. (D) Schematic representation of Mn-MOF based catalase mimic encapsulated with microfluidic microcapsules for UC therapy. Reproduced with permission from 159, Copyright © 2021 Elsevier. (E) Schematics for the relief of UC in mice after oral administration of nanozyme NiCo_2_O_4_@PVP. Reproduced with permission from 160, Copyright © 2021 WILEY.

UC will result in chronic hepatic inflammation, which is largely attributed to the fact that UC leads to the production of inflammatory macrophages, which causes the upregulation of inflammatory factors such as TNF-α and IL-6, thus allowing patients to also develop hepatobiliary disorders 156, which in turn poses a challenge to the treatment and control of UC. Fortunately, Wu *et al.* 157 established ZIF-8/siTNF-α/GSH@CaCO_3_ (ZTGC), a new drug delivery platform for the combined treatment of UC and liver injury, by coating biomineralized MOFs with calcium carbonate (CaCO_3_) (**Figure 9A**). The platform efficiently encapsulates GSH and small interfering RNA (siTNF-α), which will down-regulate the expression of inflammatory macrophages, using a biomimetic mineralization technique. The property of ZTGC to be stable in acidic conditions and to be biocompatible are conferred by the presence of calcium ions or calcium carbonate. The ZTGC technology offers a unique method of coating biomolecules, enabling NPs delivery to UC lesion sites and offering complete protection from drugs in the gastric environment. Following exposure to simulated gastrointestinal fluids, the results demonstrated that ZTGC therapy effectively reduced macrophage expression of inflammatory genes. ZTGC preferentially accumulated in mice inflamed colonic tissues, improving colonic lesions and decreasing organ inflammation to support the organism's recovery from disease, according to a bioimaging distribution analysis. Histological evaluation demonstrated that liver damage was lessened by ZTGC therapy. This research is expected to bring about a significant advancement in the development of biomolecule delivery systems for the non-pharmacological distribution of MNMs to modulate inflammation, thereby benefiting patients with UC and liver injury.

Regardless of the disease, drugs should maximize efficacy while minimizing toxic side effects. Shi *et al.* 81 combined PEI with antioxidant diselenide-bridged MONs to form nanoparticles exhibiting high cfDNA binding affinity and ROS-responsive degradation (MON-PEI) (**Figure 9B**). This is a drug-free, biosafety approach to combat UC by scavenging pro-inflammatory cfDNA and ROS. This will provide a proposal for the development of new safe and green treatments for UC. UC patients frequently face obstacles with oral drugs, including poor absorption, restricted penetration of the mucous barrier, considering the challenging undertaking of lowering excessive ROS and inflammatory cytokines. Recently, Luo *et al.* 158 developed a novel oral DDS using Janus nanomotor (Motor@M2M) covered with macrophage membrane (M2M) and loaded into sodium alginate-based hydrogel microspheres (SAM) for the targeted and effective treatment of UC without requiring for any molecular drugs (**Figure 9C**). Motor@M2M was protected from the gastric environment by SAM and smoothly released in the colon, where the MSNs functioned as a motor in this system. In addition, Motor@M2M can penetrate the colonic mucus barrier and enter the inflamed colon thanks to the propulsive force of O_2_ it generates through the catalytic consumption of local H_2_O_2_. This process scavenges ROS, attenuates hypoxia, causes the macrophage repolarization from M1 to M2, and reduces the apoptosis. The tissue targeting, overcoming intestinal barrier, and evaluating therapeutic effects of Motor@M2M@SAM-allowing inflammation neutralization, macrophage reprogramming, ROS scavenging, intestinal barrier restoration, and intestinal microbiota modulation—were systematically evaluated using a DSS-induced colitis mouse model. This novel oral delivery modality eliminates the need for molecular drugs and will provide the foundation for a 'green' therapy for UC.

#### Nanoenzyme therapy

Nanoenzyme therapy represents an emerging therapeutic strategy for treating UC by utilizing enzymatically active nanomaterials that mimic the functions of natural enzymes to achieve specific biochemical reactions, such as redox reactions, *in vivo*. Nanoenzymes with high and tunable catalytic activity, low synthesis cost, easy surface modification and good biocompatibility have attracted great research interest worldwide 163, 164. Nanoenzyme therapy has also been utilized in the treatment of many inflammatory diseases 165, 166, including UC 167. Since UC and other inflammation-related disorders are closely associated with reactive oxygen and nitrogen species, efficient antioxidants may serve as promising therapeutic agents due to their ability to scavenge free radicals. Moreover, some MNMs have also been studied to prove the existence of biological enzyme-like effects and thus used in the treatment of UC 4, 101. By using MNMs, which are similar to nano-enzymes to exert powerful biological effects, coupled with the extraordinary drug carrying and transporting capacity of MNMs, anti-inflammatory drugs can be transported to the UC lesion site and at the same time, the biocatalytic reaction of nano-enzymes can be used to specifically catalyze certain anti-inflammatory reaction processes, thus achieving the "multi-purpose" effect.

Furthermore, Chen *et al.* 159 constructed new biocompatible manganese MOF(Mn-MOF)-based catalase mimics encapsulated with microfluidic microcapsules for UC therapy (**Figure 9D**). Manganese-containing and biocompatible amino acid-based MOF microcapsules were fabricated via electrostatically driven microfluidics. The core-shell structure enables Mn-MOFs to function in the intestine, protects them from degradation in stomach fluids, ensures efficiency *in vivo* distribution, and facilitates controlled release. Upon reaching the inflamed gut, Mn-MOFs effectively scavenge ROS overproduced by neutrophils and macrophages under various GIT pH conditions, thereby shielding intestinal epithelial cells from ROS-induced damage. Notably, Mn-MOFs retain their catalase-like activity across a wide range of pH levels, particularly in near-neutral environments. This overcomes the drawback of catalase mimics, which require neutral pH for optimal performance in biological systems. We anticipate the widespread clinical usage of Mn-MOF encapsulated microcapsules in the treatment of UC and other inflammatory illnesses.

Clearance of excess ROS is significant for the treatment of UC. Recently, a novel nano-enzyme NiCo_2_O_4_@PVP was constructed by a step-by-step strategy by Zhao *et al.* 160. Notably, the presence of oxygen vacancies in NiCo_2_O_4_@PVP would help to trap oxygenated compounds, while both Co^3+^/Co^2+^ and Ni^3+^/Ni^2+^ redox pairs would provide richer catalytic sites. The obtained NiCo_2_O_4_@PVP exhibited pH-dependent multiple mimetic enzyme activities, as was to be expected. The incorporation of polyvinylpyrrolidone (PVP) endowed NiCo_2_O_4_@PVP with excellent biosafety and robust physiological stability in the gastrointestinal environment. NiCo_2_O_4_@PVP also demonstrated significant ROS scavenging activity against **·**O^2-^, H_2_O_2_, and **·**OH* in vitro*. Additionally, a DSS-induced colitis model was established to evaluate the *in vivo* anti-inflammatory properties of NiCo_2_O_4_@PVP (**Figure 9E**). In short, this work offers a promising alternative therapeutic approach for UC patients.

Previous studies have found that the onset and progression of UC is closely linked with the ROS overproduction and upregulation of transmembrane glycoprotein of CD98 168, 169. On the basis of this, Ma *et al.* 161 developed a biomimetic, pH-responsive MOF particles for the site-specific treatment of UC, designed to transport carbon nanodot-SOD nanozymes and the CRISPR/Cas9 system. As shown in (**Figure 9F**), this nanoparticle could reach the site of inflammation. After the destruction of ZIF-8 under the acidic conditions of inflammation, the nanozymes and plasmids are released. The CRISPR/Cas9 system and C-dot nanozymes reduce inflammation in UC. Under a biomimetic concept, this work combines CRISPR/Cas9 and nanozymes to develop personalized UC treatment.

**Figure 10:**
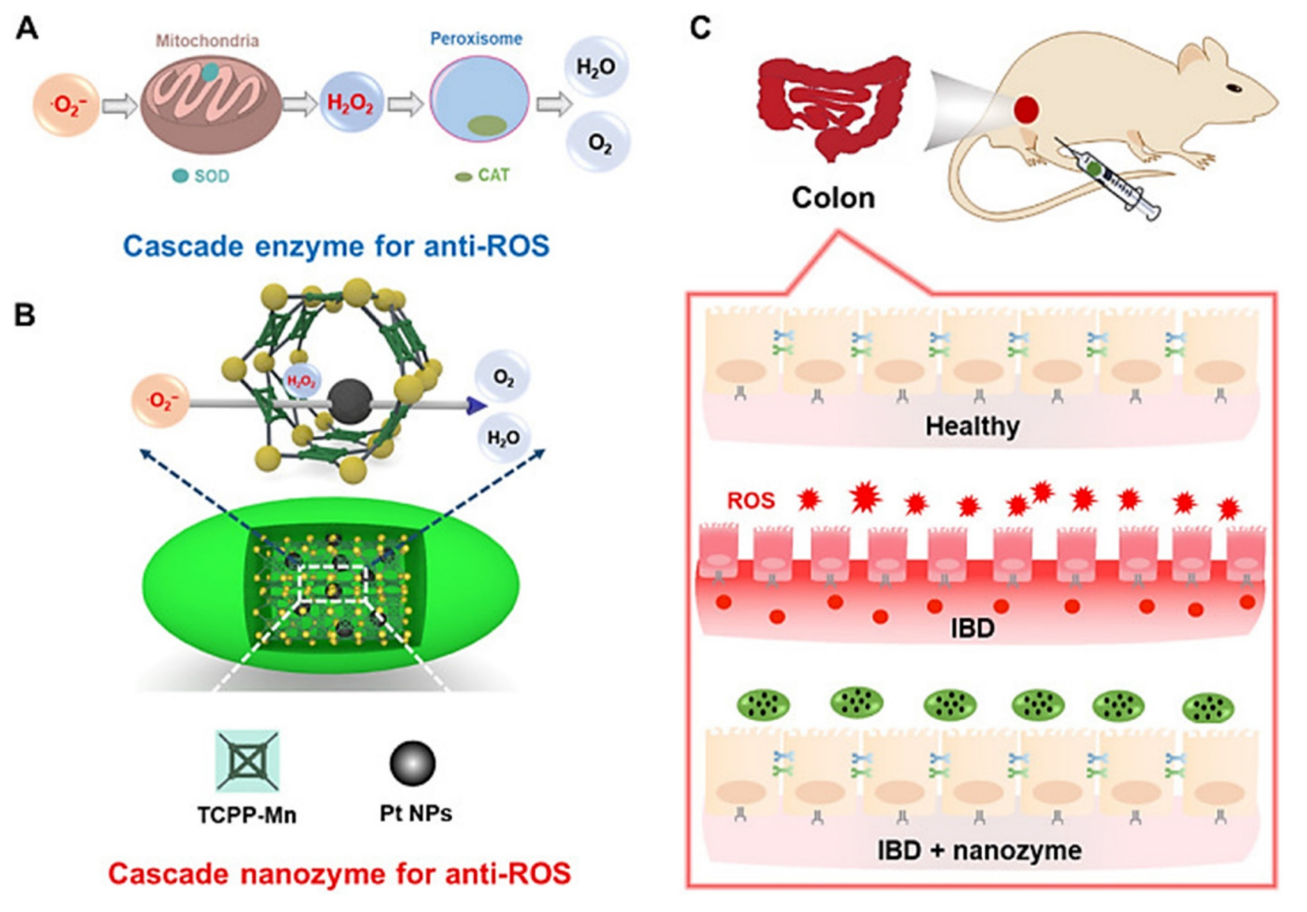
Schematic illustration of the design of an integrated nanozyme with cascade anti-ROS activity for UC therapy. Reproduced with permission from^170^, Copyright © 2020 Yufeng Liu *et al.* (A) Cellular cascade enzymes for anti-ROS. SOD and CAT enzymes have different subcellular locations and limited stability which limited their therapeutic efficacy. (B) PCN222-Mn MOF that incorporated with Pt NPs served as a cascade nanozyme for anti-ROS therapy, promoting cascade reactions through the nanoscale proximity of catalytic active sites. (C) Integrated cascade nanozyme for IBD treatment in mice.

Similarly, Li *et al.* 162 developed a gold nanoparticle-embedded cerium dioxide nano-enzyme (Au/CeO_2_) with ROS scavenging activity for the treatment of UC. Au/CeO_2_ exhibited significantly enhanced enzyme catalytic activities (SOD and CAT) compared to commercial cerium dioxide, while Au/CeO_2_@HA coated with negatively charged. Oral administration of HA significantly alleviated colonic injury in colitis rats, reduced pro-inflammatory cytokines, and promoted accumulation in inflamed colon tissues. This study provided an effective antioxidant nanotherapeutic agent for UC treatment while demonstrating a versatile approach to enhance the catalytic activity of cerium dioxide. Liu *et al.* 170 reported MOFs doped with Mn-[5,10,15,20-tetrakis(4-carboxyphenyl)porphyrinato] (TCPP) moieties, which mimic both superoxide dismutase and catalase; reducing **·**O^2-^ to H_2_O_2_, and subsequently breaking it down to water and oxygen (**Figure 9G**). These nanozyme cascade reactions can thus effectively eliminate reactive oxygen species and have been proven useful for treating two inflammatory bowel diseases-Crohn's disease and UC. Wei's team 170 developed Pt@PCN222-Mn based integrated cascade nanozyme formulation, which is essentially a nanoscale metal-organic frameworks (MOFs) material for eliminating excess ROS. Consequently, this nanozyme mimics superoxide dismutase by incorporating TCPP-a MOFs capable of converting oxygen radicals to hydrogen peroxide. Furthermore, by incorporating platinum nanoparticles that accelerate the disproportionation of hydrogen peroxide into oxygen and water, it mimicked catalase activity. The experimental results demonstrated the synergistic ROS scavenging capability of this integrated cascade nanozyme. Beyond UC, this approach has also shown promise in alleviating Crohn's disease. In addition to providing a novel approach for constructing enzyme-like cascade systems using MNMs, this work highlighted the therapeutic potential of these molecules *in vivo* for UC treatment (**Figure 10**).

In addition to the traditional MOF materials mentioned above, recent advancements in MOF-derived materials have also shown promise in the treatment of UC. Recently, Cao *et al.* 171 reported the use of artificially enzyme-modified *Bifidobacterium longum* (BL) probiotics for remodeling the healthy immune system in inflammatory bowel disease (IBD). These purely anaerobic probiotics are susceptible to oxidative destruction by ROS in IBD, which lowers therapeutic efficiency and lengthens the treatment cycle. This is because they lack antioxidant enzymes like catalase (CAT) and superoxide dismutase (SOD). To address this challenge, Cao *et al.* developed single-atom catalyst artificial enzymes (SAzymes) by pyrolyzing metal-organic-framework-encapsulated iron precursors (Fe@MOF). These SAzymes acted as guardians of the probiotics, protecting the encapsulated microorganisms from oxidative damage in inflamed environments, thus rapidly restoring barrier function and promoting a beneficial state of the gut microbiota. Furthermore, besides offering the host health advantages, BL probiotics exhibited enhanced intestinal colonization in the colon, ensuring sustained long-term antioxidant therapy of SAzymes at the disease sites. Notably, the most significant finding was that the BL@B-SA50 formulation markedly accelerated the clinical translation of artificial enzyme-armed probiotics by demonstrating robust therapeutic potential in beagle dogs with induced colitis.

## Challenges of Smart Mesoporous Nanomaterials for Treatment of UC

MNMs hold significant promise as innovative nanomaterials across various fields, including medical applications, electrochemistry, and environmental remediation. However, to translate MNMs-based smart nanoplatforms from laboratory research to clinical applications in UC, several key issues that need to be addressed beyond their functional properties.

### Similarities between the UC pathological microenvironment and other disease microenvironments

Over the past decade, the development of SNDDs has drawn extensive attention to their application in the treatment of UC. The goal of this strategy is to increase therapeutic efficacy by reducing the dosage of active drugs, minimize side effects by lowering systemic drug absorption, and further decrease the frequency of administration. For UC treatment, smart response systems mainly rely on stimuli such as low pH, hypoxia, high ROS, and specific enzymes present in the ulcerative tissue microenvironment. However, the levels of these stimuli vary significantly among different diseases and tissues. Moreover, these stimuli are not unique to ulcerative tissues, in fact, they exhibit similar characteristics in other tissues, such as the tumor microenvironment, which also shares many similar features. Therefore, the delivery system mechanisms for UC should be in line with the goal of promoting healthy tissue regeneration rather than simply mimicking pathological processes. To achieve higher targeting selectivity and lower side effects, it is necessary to improve the design of response stimuli in SNDDs to enhance their specificity for particular tissues and diseases.

### Biosafety of MNMs

The biosafety of MNMs presents a multifaceted challenge influenced by their material composition, structural design, and administration routes. Different types of MNMs exhibit distinct risks due to their unique physicochemical properties. Silica-based MNMs, while biocompatible in the short term, raise concerns about long-term organ accumulation (e.g., liver, spleen) due to non-biodegradability, potentially leading to chronic inflammation or fibrosis. Surface modifications such as PEGylation can mitigate cytotoxicity 172; however, unmodified silica nanoparticles may activate the NLRP3 inflammasome, driving pro-inflammatory cytokine release 173. MCNs, although effective for drug adsorption, generate ROS, which induce oxidative stress, DNA damage, and mitochondrial dysfunction. Inhalation risks similar to those of carbon nanotubes include pulmonary inflammation due to frustrated phagocytosis by macrophages 174. MOFs face degradation-related hazards, as metal ions (e.g., Zn²⁺, Cu²⁺) leaching into tissues can disrupt cellular homeostasis, while organic linkers may degrade into cytotoxic byproducts 175. Photocatalytic MOFs further risk ROS-mediated tissue damage under light exposure 176. Mesoporous titanium dioxide (TiO₂), though useful in photodynamic therapy, exhibits UV-induced phototoxicity and NF-κB-mediated inflammation, exacerbating conditions like colitis 177.

The biological safety of MNMs is also influenced by the route of administration. Oral delivery of MNMs may disrupt gut microbiota or epithelial integrity, exacerbating dysbiosis or leaky gut syndrome. Intravenous injection poses size-dependent hazards: smaller particles (< 10 nm) risk renal toxicity, while larger ones (> 200 nm) accumulate in the mononuclear phagocyte system 178. Inhalation of carbon-based MNMs carries risks of pulmonary fibrosis. Immune interactions vary with surface properties—positively charged MNMs often provoke stronger immune responses through opsonization and macrophage uptake, while hydrophobic surfaces may enhance unintended protein adsorption 179. Genotoxicity arises from ROS generation or direct DNA interactions (e.g., carbon MNMs intercalating genetic material), with nanoparticles < 5 nm potentially crossing the blood-brain or placental barriers, posing neurotoxic or developmental risks 180.

Addressing these concerns demands a "safe-by-design" approach, including optimizing coatings (e.g., PEG, chitosan) to reduce immunogenicity, engineering biodegradability (e.g., hydrolyzable MOFs), and tailoring size/shape for efficient clearance. Preclinical studies must prioritize long-term biodistribution, organ-specific toxicity, and immune profiling. Collaborative efforts among materials scientists, toxicologists, and regulatory agencies are critical to balance innovation with safety, ensuring MNMs realize their therapeutic potential without compromising patient health.

### Optimal design and industrial scale manufacturing of MNMs

Experts in the pharmaceutical industry should also take into account the shelf-life stability, batch-to-batch consistency, scalability and cost-effectiveness of nanomaterials. Despite the advantages of high stability and large drug loading capacity of nanomaterials, significant challenges remain in scalability and reproducibility during the development process. Moreover, as part of an emerging research field, nanomaterials have not yet demonstrated clear advantages over approved materials in the medical field, which makes pharmaceutical partners and clinicians hesitant to invest in the development of nanomaterials. Although many people believe that the "high chemical diversity, adjustable porosity, large drug loading capacity and stimulus/control drug release" characteristics of nanomaterials are highly promising in biomedical applications, especially in drug delivery, we must first assess whether these properties are the key factors influencing the clinical translation of nanomaterials in the treatment of ulcerative colitis. To that end, researchers are working to address these lingering issues. The synthesis and functionalization steps during the construction of the nano-delivery platform must be reproducible to provide valuable materials for further *in vitro* and *in vivo* testing.

Transforming the optimal design into industrial-scale production is another major challenge. The key to the commercialization of nanomaterials lies in their manufacturing economy, which is mainly determined by the price of raw materials, the complexity of the synthesis process, and the synthesis cycle. In addition, the production scale is also a factor that needs to be considered. Although medical applications usually only require a small amount of materials, this small-scale demand may lead to a significant increase in the cost per unit weight; in contrast, large-scale production helps to reduce the cost per unit weight. It is worth noting that in most laboratory-scale optimal synthesis processes, practical factors such as production time, cost control, safety and environmental impacts are often not fully considered. Moreover, how to effectively eliminate the pre-leakage problem in drug delivery systems and how to achieve precise release control of drugs in both spatial and temporal dimensions remain technical challenges that need to be addressed. Finally, the lack of clear optimal design standards also becomes a bottleneck that restricts nanomaterials from achieving the highest efficiency.

## Conclusion and Perspectives

UC, a chronic IBD, poses treatment challenges due to systemic drug toxicity and poor targeting. MNMs, with tunable porosity and functionalizable surfaces, offer innovative solutions by enabling colon-specific drug delivery. Engineered with pH-responsive polymers (e.g., Eudragit^®^) or enzyme-sensitive coatings, MNMs protect drugs like corticosteroids (budesonide) or 5-ASA from gastric degradation, releasing them directly in the inflamed colon. Preclinical studies in murine models show 60-70% reductions in inflammation compared to conventional therapies, enhanced by mucoadhesive coatings (e.g., chitosan) that prolong mucosal retention. MNMs also facilitate combination therapies, co-delivering anti-inflammatory agents (tofacitinib) and microbiome modulators (probiotics) to suppress cytokines (TNF-α, IL-6) and restore gut microbial balance. Beyond drug delivery, MNMs advance theragnostic by integrating near-infrared dyes for real-time inflammation tracking and anti-TNF-α antibodies for dual therapy-imaging capabilities.

MNMs extend to gene therapy, delivering siRNA to silence pro-inflammatory genes (NF-κB, STAT3) or mRNA encoding anti-inflammatory IL-10, achieving 80% TNF-α knockdown in preclinical models. They also target dysbiosis by selectively delivering antimicrobial peptides (cathelicidin) to pathogens while promoting beneficial bacteria (*Lactobacillus*). Immunomodulatory strategies leverage MNMs functionalized with dendritic cell-targeting ligands to induce mucosal tolerance, a promising approach in early trials. Innovations like redox-sensitive MNMs that release drugs in high-ROS environments and pore-engineered designs for hydrophobic drug loading (curcumin) bridge basic and translational science. Collaborative efforts, such as the NanoMILE project and startups like EnteraBio, are advancing MNMs into Phase II trials, while ongoing studies (e.g., NCT04640375) test ROS-scavenging polydopamine nanoparticles. Personalized MNMs tailored to biomarkers (fecal calprotectin) could enable precision dosing.

However, despite the significant potential of smart nano-therapies, several critical challenges must be addressed to facilitate clinical translation of MNMs from the laboratory. Firstly, the pathological microenvironment of UC is highly complex. Although various assistive technologies are employed in UC treatment, current methodologies have not yet achieved complete differentiation based solely on stimuli response. A deeper understanding of the unique characteristics of the UC microenvironment is essential for developing more effective therapeutic strategies. Secondly, while the FDA has classified some MNMs as 'generally safe', a comprehensive toxicology profile, particularly concerning their impact on the gastrointestinal tract, remains imperative for ensuring patient safety. Thirdly, the synthesis process for MNMs is often intricate, with poor reproducibility and high costs significantly hindering large-scale manufacturing, especially under GMP standards. Future research should focus on improving scalability and cost-effectiveness, as these factors are crucial for the successful translation and commercialization of promising MNMs candidates. Therefore, the design of MNMs-based smart nanoplatforms for UC treatment should prioritize simplicity and ease of production. Finally, biological interactions can substantially influence nanoparticle behavior and efficacy. In-depth understanding of the MNMs' physicochemical changes during UC therapy and their biological fate requires extensive and systematic investigations. Achieving safer therapeutic translations would benefit from designing MNMs with tunable and controllable rates of bioaccumulation and biodegradation. Since these characteristics influence both pharmacokinetics and biodegradation, precise control of physicochemical factors, such as particle size and surface coating, becomes essential.

In summary, MNMs hold transformative potential for UC through targeted drug delivery, microbiome modulation, and immunoregulation. While preclinical successes are promising, translational progress hinges on resolving oral toxicity, manufacturing scalability, and regulatory alignment. Collaborative efforts between academia and industry, coupled with patient-centric design, will accelerate MNMs from bench to bedside, offering hope for UC patients resistant to conventional therapies.

## Abbreviations

UC: Ulcerative colitis
MNMs: Mesoporous nanomaterials
WHO: World Health Organization
SNDDSs: Smart nano-drug delivery systems
GIT: gastrointestinal tract
DDSs: drug delivery systems
IUPAC: International Union of Pure and Applied Chemistry
MSNs: Mesoporous silica nanoparticles
MOFs: Metal-organic frameworks
MPDA: Mesoporous polydopamine
ROS: Reactive Oxygen Species
TNF-α: tumor necrosis factor-α
IL: interleukins
MUC-2: mucin-2
Th: T-cell
TLR: Toll-like receptors
ALPs: alkaline phosphatases
HNE: human neutrophil elastase
MMPs: matrix metalloproteinases
IECs: intestinal epithelial cells
M cells: Microfold cells
BCS: Biopharmaceutical Classification System
5-ASA: 5-Aminosalicylic Acid
KIT: Korean Institute of Science and Technology
IBN: Institute of Bioengineering and Nanotechnology
2D: two-dimensional
3D: three-dimensional
SPL: succinylated ε-polylysine
S1: safranin O
S2: hydrocortisone
PEI: polyethylenimine
cf-DNA: cell-free DNA
DSS: Dextran Sulfate Sodium
TNBS: 2,4,6-Trinitrobenzenesulfonic acid
PEG: polyethylene glycol
FA: folic acid
DAPT: γ-secretase inhibitor
MONs: mesoporous organosilica nanoparticles
Dex: dexamethasone
SNOs: S-nitrosothiols
IRMOFs: Isoreticular MOFs
ZIFs: Zeolitic imidazolate frameworks
PCNs: Porous coordination networks
MIL: Materials institute Lavoisier
PCPs: Porous coordination polymers
UiO: University of Oslo
CD-MOFs: Cyclodextrin-based metal organic frameworks
POST-n: Pohang University of Science and Technology
NU: Northwestern University
NOTT-n: University of Nottingham
DUT-n family: Dresden University of Technology
CAU-n family: Christian-Albrechts University
HKUST-n: Hong Kong University of Science and Technology
siRNAs: Small interfering RNAs
CDF: cyclodextrin metal-organic framework
Res: resveratrol
Res-CDF in MPs: metal-organic framework encapsulated with resveratrol Res doped into natural polysaccharide hydrogel microspheres
BL: *Bifidobacterium longum*
IBD: inflammatory bowel disease
CAT: catalase
SOD: superoxide dismutase
SAzymes: single-atom catalyst artificial enzymes
Fe@MOF: metal-organic-framework-encapsulated iron precursors
PDA: Polydopamine
MP: mesoporous polymers
MPDA-siRNA@CaP: a ROS scavenging and gene interference therapeutic platform
CaP: calcium phosphate
PAA: polyacrylic acid
SAP: sulfasalazine pyridine
PAA@MPDA-SAP NPs: sulfasalazine pyridine was loaded into MPDA NPs
LBL: layer-by-layer
CO@MPDA: MPDA NPs loaded with carbon monoxide precursors
MCNs: Mesoporous carbon nanoparticles
MDC: *Musca domestica* cecropin
MCN: mesoporous cerium oxide nano-enzymes
GPx: Glutathione peroxidase
TK: thioketal
TCOF: frameworks containing thioketal
CCOF: covalent cross-linked frameworks
TCD: DEX loaded in TCOF
CCD: DEX loaded in CCOF
CD: cyclodextrin
CeNPs: ceria nanoparticles
ICANs: inflamed colon-targeted nanotherapeutics
Ce-MOF@PSS: oxidation-responsive metal-organic framework material
COX-2: cyclooxygenase-2
CS: chitosan
HMSS-N=N-CS: enzyme-responsive colon-specific delivery system of biodegradable CS via cleavable azobonds
DOX: doxorubicin
HMSS: hollow mesoporous silica spheres
Trp: tryptophan
AHR: aryl hydrocarbon receptor
CHI: chitosan
HA: hyaluronic acid
Azo: azobenzene
CB[8]: cucurbit[8]uril
(*EcN*): *Escherichia coli* strain Nissle 1917
EM: membrane
SeM: diselenide-bridged MSNs
SeM@EM: mesoporous nanosystems responsive to microbial organisms
Res-CDF in MPs: Res-CDF doped into microspheres
GMA: glycidyl methacrylate
MESL: mesalamine
M1: phenotype
FRs: folate receptors
DSMSNs: drug delivery vehicle of organosilica nanoparticles
CR: Cux-RhodamineB
HPMCAS: hydroxypropyl methylcellulose acetate succinate
CMOF: CD-MOF
Qu: quercetin
TPP: triphenyl phosphine bromide
QT): Qu-TPP precursor complex
HAS: hyaluronic acid shell
HUR: HF@UiO-CR
S2: MSNs capped with hydrolyzed starch
HA-CsT@RH: CD-supramolecular nanoparticles
RH: rhein
PepT1: peptide transporter protein 1
ZTGC: ZIF-8/siTNF-α/GSH@CaCO_3_
siTNF-α: small interfering RNA
Motor@M2M: Janus nanomotor
M2M: macrophage membrane
SAM: sodium alginate hydrogel microspheres
PVP: polyvinylpyrrolidone
Cas9: protein 9
CRISPR: clustered regularly interspaced short palindromic repeats
Cur: curcumin
CF@EM: Cur-Fe nanozyme was integrating into the *EcN* membrane
EM: *EcN* membrane
ALG: calcium alginate
LA: alpha-lipoic acid
CSO: chitosan oligosaccharide
Cu_5.4_O@SAG: dual-pH/H_2_S responsive nanoplatform
Au/CeO_2_: gold nanoparticle-embedded cerium dioxide nano-enzyme
TCPP: Mn-[5,10,15,20-tetrakis(4-carboxyphenyl)porphyrinato]
Pt@PCN222-Mn: Pt NPs inside PCN222-Mn MOF
TiO₂: Mesoporous titanium dioxide
GMP: Good Manufacturing Practice
